# Multiplex Identification of Human Papillomavirus 16 DNA Integration Sites in Cervical Carcinomas

**DOI:** 10.1371/journal.pone.0066693

**Published:** 2013-06-18

**Authors:** Bo Xu, Sasithorn Chotewutmontri, Stephan Wolf, Ursula Klos, Martina Schmitz, Matthias Dürst, Elisabeth Schwarz

**Affiliations:** 1 Research Program Infection and Cancer, DKFZ, Heidelberg, Germany; 2 Genomics and Proteomics Core Facility, DKFZ, Heidelberg, Germany; 3 Department for Gynecology, Jena University Hospital, Jena, Germany; Karolinska Institutet, Sweden

## Abstract

Cervical cancer is caused by high-risk human papillomaviruses (HPV), in more than half of the worldwide cases by HPV16. Viral DNA integration into the host genome is a frequent mutation in cervical carcinogenesis. Because integration occurs into different genomic locations, it creates unique viral-cellular DNA junctions in every single case. This singularity complicates the precise identification of HPV integration sites enormously. We report here the development of a novel multiplex strategy for sequence determination of HPV16 DNA integration sites. It includes DNA fragmentation and adapter tagging, PCR enrichment of the HPV16 early region, Illumina next-generation sequencing, data processing, and validation of candidate integration sites by junction-PCR. This strategy was performed with 51 cervical cancer samples (47 primary tumors and 4 cell lines). Altogether 75 HPV16 integration sites (3′-junctions) were identified and assigned to the individual samples. By comparing the DNA junctions with the presence of viral oncogene fusion transcripts, 44 tumors could be classified into four groups: Tumors with one transcriptionally active HPV16 integrate (n = 12), tumors with transcribed and silent DNA junctions (n = 8), tumors carrying episomal HPV16 DNA (n = 10), and tumors with one to six DNA junctions, but without fusion transcripts (n = 14). The 3′-breakpoints of integrated HPV16 DNA show a statistically significant (p<0.05) preferential distribution within the early region segment upstream of the major splice acceptor underscoring the importance of deregulated viral oncogene expression for carcinogenesis. Half of the mapped HPV16 integration sites target cellular genes pointing to a direct influence of HPV integration on host genes (insertional mutagenesis). In summary, the multiplex strategy for HPV16 integration site determination worked very efficiently. It will open new avenues for comprehensive mapping of HPV integration sites and for the possible use of HPV integration sites as individualized biomarkers after cancer treatment of patients for the early diagnosis of residual and recurrent disease.

## Introduction

Persistent infection with carcinogenic human papillomavirus (HPV) is the essential basis for development of cervical cancer [1], one of the most common cancers in women worldwide [2], [3]. From the twelve mucosotropic high-risk HPV types (hr-HPV) classified as “carcinogenic to humans” [4], HPV16 is by far the most prevalent and most carcinogenic type responsible for more than 50% of all cervical cancer cases worldwide, followed by HPV18 (about 20% of cervical cancer cases) and less prevalent hr-HPV types [5]–[7]. Most cervical hr-HPV infections are transient and cleared within 1–2 years. Long-term viral persistence is established in about 10% of the infection cases, and only some of the persistent hr-HPV infections will progress to precancer lesions and eventually to cancer [1]. HPV16 is present in the human population in many different molecular variants, which have been grouped into five phylogenetic clusters based on their original geographic distribution [8]. HPV16 variants differ in their carcinogenic potential and other transformation-linked properties [9], [10].

The viral oncogenes E6 and E7 become constitutive components of the host cells by persistent hr-HPV infection. Their protein products inactivate the major cellular tumor suppressors p53 and pRB, and interact in addition with a plethora of other cellular proteins [11]–[13]. The HPV life cycle and viral gene expression patterns are severely disturbed in the course of cervical carcinogenesis [14]. Deregulated constitutive expression of E6 and E7 is the key event for malignant progression, combined with additional alterations of viral and cellular genes and pathways [15], [16].

Integration of hr-HPV DNA into the host genome can be a driver mutation in cervical carcinogenesis, associated with progression and invasiveness [17], [18]. The prevalence of integrated hr-HPV DNA increases substantially with the severity of the lesions, reaching 100% in HPV18-induced cervical cancer cases [19]–[22]. A subset of HPV16-positive invasive cervical carcinomas, however, maintains viral DNA only as episomes indicating that integration-associated and episome-associated pathways of HPV16-induced cervical carcinogenesis might exist [23], [24].

**Figure 1:**
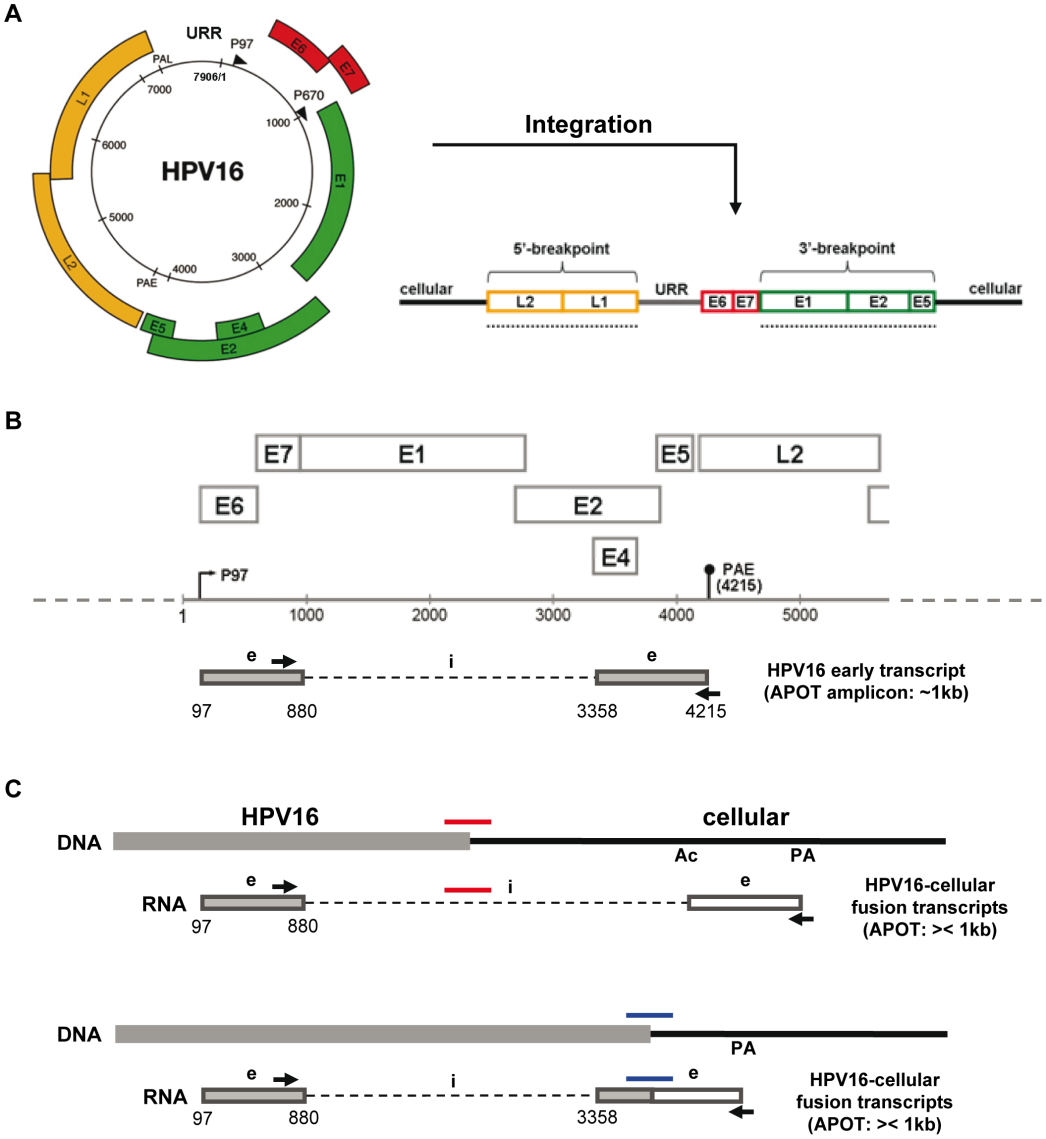
Genomic structure and transcription of episomal and integrated HPV16 DNA. (A) The circular HPV16 genome becomes linearized and inserted into the host cell genome upon integration. The breakpoints of integrated HPV16 DNA can be located anywhere in the L2-L1 region (5′ breakpoint) and E1-E2-E5 region (3′ breakpoint), respectively. The breakpoint regions are indicated by dotted underlines. The circular HPV16 genome is reproduced with slight modifications with permission, from Doorbar, (2006), (Clinical Science), (110), (525–541). © the Biochemical Society. (B) In case of episomal HPV16 DNA, early transcription is initiated at the early promoter P97 and terminated at the early poly-A signal (PAE at pos. 4215). The early transcript shown contains two exons (e) with ORFs for E6, E7, E1?E4 and E5. An intron (i) of 2477 nucleotides is removed by splicing at the indicated donor and acceptor positions. Amplification of HPV16 oncogene transcripts by the APOT assay gives rise to a constant-size RT-PCR amplicon of ∼1 kb [21]. The APOT primers are indicated by the arrows. (C) Early transcription from integrated HPV16 DNA will lead to HPV16-cellular fusion transcripts, because the viral PAE signal is missing and instead a cellular poly-A signal (PA) is adopted. If the 3′ breakpoint is located upstream of the splice acceptor at position 3358, an alternative cellular splice acceptor (Ac) will be used, and the HPV16-cellular DNA junction sequence (red bar) will be spliced out as part of a viral-cellular intron (upper part). If the 3′ breakpoint is located downstream of the splice acceptor 3358, the HPV16-cellular DNA junction sequence (blue bar) will remain as part of a viral-cellular exon, and the DNA and RNA junction sequences are colinear (lower part). The HPV16-cellular fusion transcripts are amplified in APOT assays as RT-PCR products that are shorter or longer than the ∼1-kb amplicon derived from episomal HPV16 [21].

Integration converts the circular HPV genome into a linear truncated DNA, in which the upstream regulatory region (URR) and the E6/E7 oncogenes are always retained intact (Figure 1A) [22], [25]. Besides the integrated monomeric forms, head-to-tail concatemers of full-length HPV genomes flanked by truncated copies also exist, exemplified by the cervical cancer cell line CaSki [26], [27]. Transcription initiated at the HPV early promoter traverses the 3′ integration site into the flanking cellular sequences, giving rise to spliced viral-cellular fusion transcripts that are important for constitutive deregulated expression of the E6/E7 oncogenes (Figure 1B and 1C) [21], [22], [28]–[31].

Integrated HPV DNA usually shows disruption or complete deletion of the E1 or E2 gene, with a consequence of functional inactivation. The E1 gene encodes the HPV-specific helicase essential for initiation of viral DNA replication. The E2 gene encodes a multifunctional regulatory protein involved in regulation of viral transcription, initiation of viral DNA replication and maintenance of the viral DNA episome. Loss of the E1/E2 expression abrogates the E2-mediated repression of E6/E7 transcription from integrated HPV DNA [32], increases the efficiency of HPV-induced immortalization of primary human keratinocytes [33], and is associated with poor prognosis of cervical cancer as well as low disease-free survival rate [34], [35].

HPV DNA integration occurs into various regions of the human genome, with certain preferences for transcribed regions and common fragile sites [36]–[38]. Many HPV integration sites are located within known or predicted cellular genes [28]. Inactivation of tumor suppressor genes or activation of proto-oncogenes might be a direct consequence of HPV DNA integration. Examples for such scenarios have been reported, including the *MYC* proto-oncogene [36]–[42] and the potential tumor suppressor gene *ZBTB7C* (*APM-1*) [43] and *CASZ1* [44]. It is a matter of ongoing debate whether and to which extent HPV-induced insertional mutagenesis of cellular genes contributes to cervical carcinogenesis [28], [30], [38], [45].

Sequence determination of viral-cellular junctions gives direct proof of HPV integration and allows precise localization of the chromosomal target sites. However, this is also a difficult task because the integration breakpoints of both the viral and cellular genome are different in all samples. In the past, junction sequences were determined in clones isolated from genomic DNA or cDNA libraries [22], [26], [29]. Later, PCR-based approaches for DNA junction analysis were developed including restriction-site PCR (RS-PCR) [37], [46], [47], detection of integrated papillomavirus sequences by ligation-mediated PCR (DIPS-PCR) [48], and the restriction-ligation-inverse PCR (rli-PCR) [49]. But these PCR methods are quite laborious and not sensitive enough, therefore limiting the broad application of them. For RNA junction analysis, the amplification of papillomavirus oncogene transcripts (APOT) RT-PCR assay can distinguish episome-derived viral transcripts (Figure 1B) from integrate-derived viral-cellular fusion transcripts (Figure 1C) [21], [30]. Until now, viral-cellular junctions from more than 300 cervical precancer and cancer samples have been characterized [28], [38], [50]. Development of more efficient methods would facilitate a comprehensive mapping of HPV integration sites to gain deeper insight into cervical carcinogenesis. Furthermore, efficient determination of HPV integration sites would allow their use as individualized markers in cervical cancer screening and in the follow-up of patients for the early detection of recurrent disease and metastasis.

Here we report the design and application of an innovative strategy for the simultaneous, nucleotide-level determination of HPV16 DNA integration sites in multiple cervical cancer samples. The multiplex strategy takes advantage of novel methods for sample preparation and next-generation sequencing (NGS). In a pooled mixture of DNA samples from about 50 primary cervical carcinomas and carcinoma-derived cell lines, more than 70 HPV16-cellular junction sequences could be identified in one NGS experiment and assigned by junction-specific PCR to the individual samples. The power of NGS was concomitantly employed to determine the HPV16 E6 variant sequences for all carcinoma samples. The efficient performance opens new avenues in the future for HPV16 integration site analysis in large numbers of cancer and precancer samples.

## Results

Identification of HPV DNA integration sites in the human genome is an elaborate task due to the unique combination of viral and cellular breakpoints in every single case. Exploiting the high capacity of NGS, we have designed and applied a multiplex strategy for HPV16 integration site determination, named TEN16 for “Tagging, Enrichment and Next-generation sequencing of HPV16”.

### TEN16 Assay Design

**Figure 2:**
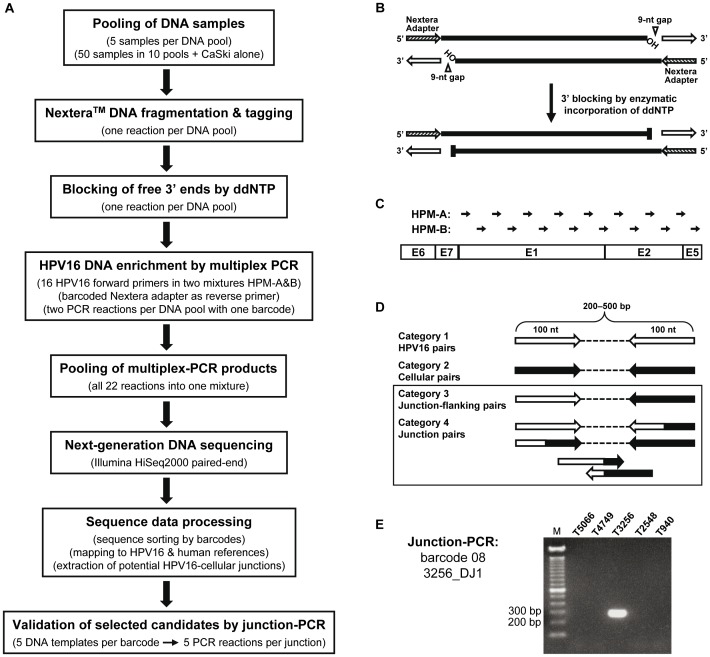
The TEN16 strategy for simultaneous determination of HPV16 DNA integration sites in multiple samples. (A) The TEN16 workflow. Details of the individual steps are given in parentheses. (B) Structure of DNA fragments produced by Nextera reaction and blocking of the DNA 3′-ends. DNA fragmented by Nextera transposition carries a 19-nt universal adapter (striated arrow; referred to as Nextera adapter) covalently coupled to the 5′-end of each strand, whereas a 9-nt gap separates the 3′-end from the complementary oligonucleotide (open arrow) [52]. In a PCR using Nextera DNA fragments as templates, DNA polymerase will repair the 9-nt gap. This will lead to whole-genome amplification if the Nextera adapter is used as a primer. For this reason, a blocking reaction with ddNTP was conceived to make the 3′-OH groups inaccessible for gap repair to reduce the amplification primed by Nextera adapter alone. (C) Enrichment of the HPV16 early region by multiplex PCR. Sixteen HPV16 forward primers assembled in two mixtures (HPM-A and -B) are used to cover the E1-E2-E5 region, where the viral 3′ breakpoints can occur. The approximate primer locations are marked by arrows. (D) Categories of HiSeq2000 read pairs after sorting and mapping. In category 1 both reads of the same pair contain HPV16 sequences (open arrow) only, and in category 2 cellular sequences (filled arrow) only. Category 3 contains pairs with one HPV16 read and a cellular mate. Category 4 contains pairs with one junction read (combined arrow) plus an HPV16, a cellular, or a junction mate. Categories 3 and 4 are important for HPV16 integration site determination. (E) Validation of potential HPV16-cellular junctions by junction-specific PCR. The five DNA samples sharing the same barcode are used individually as PCR templates, and only one of them should be positive for the junction being tested. Shown here is the junction-PCR for the five DNA samples of barcode 08 and the positive result for 3256_DJ1.

The TEN16 strategy is a multistep procedure including as essential steps (i) Nextera™ *in vitro* transposition for fragmentation and adapter tagging of the genomic DNA of HPV16-positive tumor samples; (ii) HPV16 DNA enrichment by multiplex PCR with HPV16 forward primers and barcoded Nextera adapter as reverse primer; (iii) Illumina NGS; (iv) data processing for sequence sorting and mapping; and (v) validation of HPV16-cellular junction sequences by junction-specific PCR. The complete workflow is outlined in Figure 2A.

Nextera™ *in vitro* transposition is a novel sample preparation approach for whole-genome NGS [51]. In a single reaction, the sample DNA is randomly fragmented, and the fragments are universally tagged with a specific Nextera adapter sequence (the transposon end) at the 5′-ends (Figure 2B). This feature makes the Nextera technique especially suitable for PCR enrichment of HPV16-cellular junction sequences from the complex human genome. The transposition reaction creates double-stranded DNA fragments with transposon-tagged 5′-ends and free 3′-ends upstream of a 9-nt single-stranded gap [52]. To facilitate HPV16 DNA enrichment and to suppress the unwanted PCR amplification of pure genomic DNA, the standard Nextera workflow was modified by blocking the free 3′-ends with ddNTP after DNA fragmentation/tagging and transposase removal (Figure 2B).

The Illumina HiSeq2000 NGS system was employed for DNA sequencing using the paired-end method with read length of 2×100 nucleotides (nt). Before sequencing, the enrichment of HPV16-containing sequences from the whole genomic DNA was achieved by multiplex PCR reactions. The focus was put on the functionally important and potentially transcribed 3′ breakpoints of integrated HPV16 DNA. To detect all possible 3′ breakpoints, 16 forward primers were selected which completely cover the HPV16 E1-E2-E5 region of 3.2 kb in length (Figure 2C). The primer sequences are given in Table S1. With an estimated yield per lane of 100 million read pairs by HiSeq2000 sequencing, it was calculated that the simultaneous analysis of 50 samples in one TEN16 experiment would result in a sequencing depth of 125,000 read pairs per sample per HPV16 primer. This should be sensitive enough to determine viral-cellular junctions against the background of pure HPV16 sequences.

To simplify the whole experimental procedure and to reduce the per-sample cost of Nextera, 50 DNA samples were partitioned into ten pools (five samples each) before the Nextera reaction and then processed in parallel. Multiplex PCR for HPV16 DNA enrichment was performed for each pool in two reactions with two HPV16 forward primer mixtures HPM-A and -B (eight primers each), respectively. The Nextera adapter was used as reverse primer equipped at the 5′-end with a unique 5-nt barcode for each pool (Table S2), necessary for later sequence sorting. Products of all PCR reactions were mixed together and prepared for HiSeq2000 sequencing.

### TEN16 Performance of HPV16 Integration Site Determination

In the TEN16 pilot study, 51 DNA samples including 47 fresh-frozen cervical carcinomas and four cervical cancer cell lines (SiHa, CaSki, MRI-H186, MRI-H196) were investigated. Fifty of them were premixed into ten pools as described above. CaSki DNA was prepared separately to assess the sensitivity of TEN16, because every CaSki cell harbors as much as 600 copies of integrated full-length, concatemeric HPV16 DNA [26], [53].

A total of 106.3 million sequence read pairs were generated from a single lane of HiSeq2000. Almost 72 million read pairs were identifiable and sorted by barcodes (no mismatch allowed in the barcode and primer sequences). Over 70 million read pairs represented the TEN16 DNA library, and the rest belonged to the HPV16 E6 variant analysis (described later). After mapping with HPV16 and human reference sequences, the sorted TEN16 read pairs were split into four categories (Figure 2D). On average per barcode, 53% contained pure HPV16 sequences (category 1), 42% contained pure cellular sequences (category 2), 0.05% were read pairs flanking HPV16-cellular junctions (category 3), 0.25% contained a chimeric sequence in at least one read (category 4), and 4.7% were unmapped sequences.

The data processing gave rise to about 200,000 read pairs in categories 3 and 4. Visual inspection of the sequences revealed that many potential junctions were represented by only one or a few read pairs. Because it was unfeasible to validate all the candidates by junction-PCR, a cutoff value of 15 read pairs per junction was chosen. With this criterion, 76 potential junctions were selected as the most promising candidates for validation. Among them, 67 junctions were confirmed to be authentic by junction-PCR and Sanger sequencing, and were assigned to the individual tumors or cell lines (Figure 2E). Nine junctions could not be assigned to any sample in the respective barcode. They were further analyzed individually with all DNA pools to check for the possibility of wrong barcode assignment. Since all reactions were negative (data not shown), it was concluded that the nine junctions are most likely PCR artifacts during HPV16 DNA enrichment.

For CaSki, three novel junctions were identified by TEN16 with >15 read pairs each. The previously known junction [54] was detected by searching again through the TEN16 sequence library, but only with three read pairs (CS_DJ1 in Table 1). Additional junctions were discovered later while comparing DNA junction/RNA junction sequences (see next chapter). Taken together, 75 HPV16-cellular DNA junctions were identified and validated in the TEN16 study (Table 1 and Table S3). Distribution of the read pair numbers for all junctions is shown in Figure S1.

**Table 1:** HPV16-cellular DNA junctions validated by junction-PCR.

		HPV16	Cellular Sequence				Cellular Gene			Accession
DJ1)	Sample	breakpoint (ORF)	breakpoint	map	strand	u/r2)	name	t/d3)	orientation4)	Number
2319_DJ3a)	T2319	910 (E1)	10697545	1p36.22	–	u	*CASZ1*	t	same	HE984526
2319_DJ2 a)	T2319	3115 (E2)	10699282	1p36.22	–	u	*CASZ1*	t	same	HE984525
2319_DJ1 a)	T2319	4338 (L2)	10707431	1p36.22	+	u	*CASZ1*	t	opposite	HE984524
0892_DJ2 b)	T892	2886 (E2)	27869153	2p23.2	–	r (AluSx)	*GPN1*	t	opposite	HE984511
0892_DJ1	T892	2970 (E2)	27869352	2p23.2	+	u	*GPN1*	t	same	HE984510
3719_DJ1	T3719	2823 (E2)	34896821	2p22.3	–	r (L1M1_5)	*no gene*			HE984545
2882_DJ1	T2882	3596 (E2)	146512049	2q22.3	–	u	*no gene*			HE984537
2085_DJ1	T2085	2774 (E1/E2)	149157969	2q23.1	+	u	*MBD5*	t	same	HE984518
1875_DJ1	T1875	3010 (E2)	201823720	2q33.1	+	u	*ORC2*	t	opposite	HE984516
0841_DJ1	T841	2412 (E1)	206103492	2q33.3	–	r (AluSq)	*PARD3B*	t	opposite	HE984505
3987_DJ1	T3987	3489 (E2)	212251450	2q34	+	u	*ERBB4*	t	opposite	HE984548
2317_DJ1	T2317	3667 (E2)	230045578	2q36.3	+	u	*PID1*	t	opposite	HE984522
5234_DJ1	T5234	2574 (E1)	49319411	3p21.31	–	r (AluSc)	*USP4*	t	same	HE984565
2548_DJ1	T2548	2868 (E2)	60486966	3p14.2	–	u	*FHIT*	t	same	HE984528
2548_DJ2	T2548	1921 (E1)	60535200	3p14.2	+	u	*FHIT*	t	opposite	HE984529
0018_DJ1	T18	3048 (E2)	169306956	3q26.2	+	u	*MECOM*	t	opposite	HE984501
3256_DJ1	T3256	1642 (E1)	182088476	3q26.33	+	u	*ATP11B*	d	same	HE984539
4426_DJ1 c)	T4426	2080 (E1)	190056682	3q28	–	u	*CLDN1*	d	same	HE984552
3966_DJ1 d)	T3966	2990 (E2)	74540049	4q13.3	+	u	*IL8*	d	same	HE984547
3719_DJ2	T3719	2615 (E1)	78848346	4q21.1	–	u	*MRPL1*	t	opposite	HE984546
4024_DJ2 c)	T4024	1841 (E1)	100450200	4q23	–	u	*C4orf17*	t	opposite	HE984550
2231_DJ1 e)	T2231	1030 (E1)	191040400	4q35.2	–	r (MER31_I)	*DUX4L2*	d	opposite	HE984521
2592_DJ1	T2592	1948 (E1)	4583408	5p15.32	–	u	*no gene*			HE984534
5189_DJ1	T5189	3286 (E2)	25574739	5p14.1	+	u	*no gene*			HE984562
3576_DJ1	T3576	2350 (E1)	44184762	6p21.1	+	u	*SLC29A1*	d	same	HE984544
CS_DJ1 f)	CaSki	3729 (E2)	45659125	6p12.3	–	u	*RUNX2*	d	opposite	HE984566
4977_DJ1	T4977	2143 (E1)	126782491	6q22.32	–	r (LTR)	*CENPW*	d	opposite	HE984559
4977_DJ2	T4977	3059 (E2)	28835005	7p15.1	–	u	*CREB5*	t	opposite	HE984560
5066_DJ2	T5066	1813 (E1)	61969363	7q11.21	–	r (ALR1)	*no gene*			HF559481
5189_DJ2	T5189	2627 (E1)	111272354	8q23.2	+	r (L1)	*no gene*			HE984563
1509_DJ1	T1509	2686 (E1)	113482518	8q23.3	+	u	*CSMD3*	t	opposite	HE984513
5189_DJ3	T5189	2353 (E1)	128396523	8q24.21	+	r (AluY)	*POU5F1B*	d	same	HE984564
MH186_DJ1	MRI–H186	1224 (E1)	128675817	8q24.21	+	u	*MYC*	d	same	HE984570
MH186_DJ2	MRI–H186	2754 (E1)	128746603	8q24.21	+	u	*MYC*	d	same	HE984571
4601_DJ1	T4601	999 (E1)	13858043	9p23	+	u	*LINC00583*	d	same	HE984553
0186_DJ1	T186e	2590 (E1)	26947928	9p21.2	–	u	*IFT74*	t	opposite	HE984503
1509_DJ2	T1509	1228 (E1)	33980798	9p13.3	+	r (AluJb)	*UBAP2*	t	opposite	HE984514
4793_DJ3 g)	T4793	1226 (E1)	126291635	9q33.3	+	u	*DENND1A*	t	opposite	HE999548
CS_DJ2	CaSki	1973 (E1)	11742450	10p14	–	u	*USP6NL*	d	same	HE984567
3256_DJ2	T3256	2344 (E1)	100058840	10q24.2	–	u	*LOXL4*	d	same	HE984540
3256_DJ3	T3256	2612 (E1)	100058865	10q24.2	+	u	*PYROXD2*	d	opposite	HE984541
MH196_DJ1	MRI–H196	3858 (E5)	47967861	11p11.2	+	u	*PTPRJ*	d	same	HE984572
3427_DJ1	T3427	1794 (E1)	12233422	12p13.2	–	r (AluSg)	*BCL2L14*	t	opposite	HE984542
0841_DJ2	T841	1953 (E1)	73677253	13q22.1	–	u	*KLF5*	d	opposite	HE984506
4046_DJ1	T4046	2146 (E1)	73999476	13q22.1	–	u	*KLF5*	d	opposite	HE984551
SH_DJ1	SiHa	3133 (E2)	74087562	13q22.1	–	u	*KLF5*	d	opposite	HE984573
0841_DJ3	T841	3281 (E2)	74226231	13q22.2	+	u	*KLF12*	d	opposite	HE984507
2209_DJ1	T2209	2637 (E1)	74255979	13q22.2	+	u	*KLF12*	d	opposite	HE984520
1907_DJ1	T1907U	2412 (E1)	62135042	14q23.2	+	u	*HIF1A*	d	same	HE984517
0182_DJ1	T182e	2725 (E1)	58836257	15q22.2	+	u	*LIPC*	t	same	HE984502
2317_DJ2	T2317	1028 (E1)	90729527	15q26.1	–	u	*SEMA4B*	t	opposite	HE984523
2085_DJ2	T2085	2198 (E1)	11918118	16p13.13	–	u	*BCAR4*	t	same	HE984519
3427_DJ2	T3427	2461 (E1)	19657318	17p11.2	+	u	*ULK2*	d	opposite	HE984543
2707_DJ1	T2707	3564 (E2)	37818061	17q21.31	+	u	*STARD3*	t	same	HE984535
2707_DJ2	T2707	2970 (E2)	37862307	17q21.31	–	u	*ERBB2*	t	opposite	HE984536
1686_DJ1	T1686	1354 (E1)	57908866	17q23.2	–	u	*VMP1*	t	opposite	HE984515
4749_DJ1	T4749	2564 (E1)	1493334	18p11.32	+	r (L1)	*no gene*			HE984554
4749_DJ2	T4749	1027 (E1)	1506143	18p11.32	–	u	*LINC00470*	d	same	HE984555
4749_DJ3	T4749	3337 (E2)	34242009	18q12.2	+	u	*FHOD3*	t	same	HE984556
2548_DJ3	T2548	2019 (E1)	963052	19p13.3	–	u	*ARID3A*	t	opposite	HE984530
2548_DJ4	T2548	3890 (E5)	2080380	19p13.3	–	u	*MOB3A*	t	same	HE984531
2967_DJ1	T2967	2132 (E1)	19610972	19p13.11	+	u	*GATAD2A*	t	same	HE984538
0186_DJ2	T186e	3897 (E5)	30498221	19q11	+	u	*URI1*	t	same	HE984504
5066_DJ1	T5066	2470 (E1)	11159613	20p12.2	+	u	*no gene*			HE984561
2548_DJ5	T2548	2406 (E1)	14886856	20p12.1	+	r (AluSz)	*MACROD2*	t	same	HE984532
2548_DJ6	T2548	3888 (E5)	32116640	20q11.22	+	u	*CBFA2T2*	t	same	HE984533
2349_DJ1	T2349	2949 (E2)	7521372	Xp22.31	–	u	*STS*	d	opposite	HE984527
4024_DJ1	T4024	3018 (E2)	17517026	Xp22.13	+	u	*NHS*	t	same	HE984549
0841_DJ4	T841	3413 (E2)	24333382	Xp22.11	–	u	*FAM48B2*	d	same	HE984508
0841_DJ5	T841	1703 (E1)	24564835	Xp22.11	–	u	*PDK3*	d	opposite	HE984509
0940_DJ1	T940	1529 (E1)	124850317	Xq25	+	u	*DCAF12L2*	d	opposite	HE984512
CS_DJ3	CaSki	975 (E1)	144778296	Xq27.3	+	u	*SLITRK2*	d	same	HE984568
CS_DJ4	CaSki	2987 (E2)	144789749	Xq27.3	+	u	*SLITRK2*	d	same	HE984569
4793_DJ1 h)	T4793	1932 (E1)				r (GGAAT)				HE984557
4793_DJ2 h)	T4793	3881 (E5)				r (GGAAT)				HE984558

**Figure 3:**
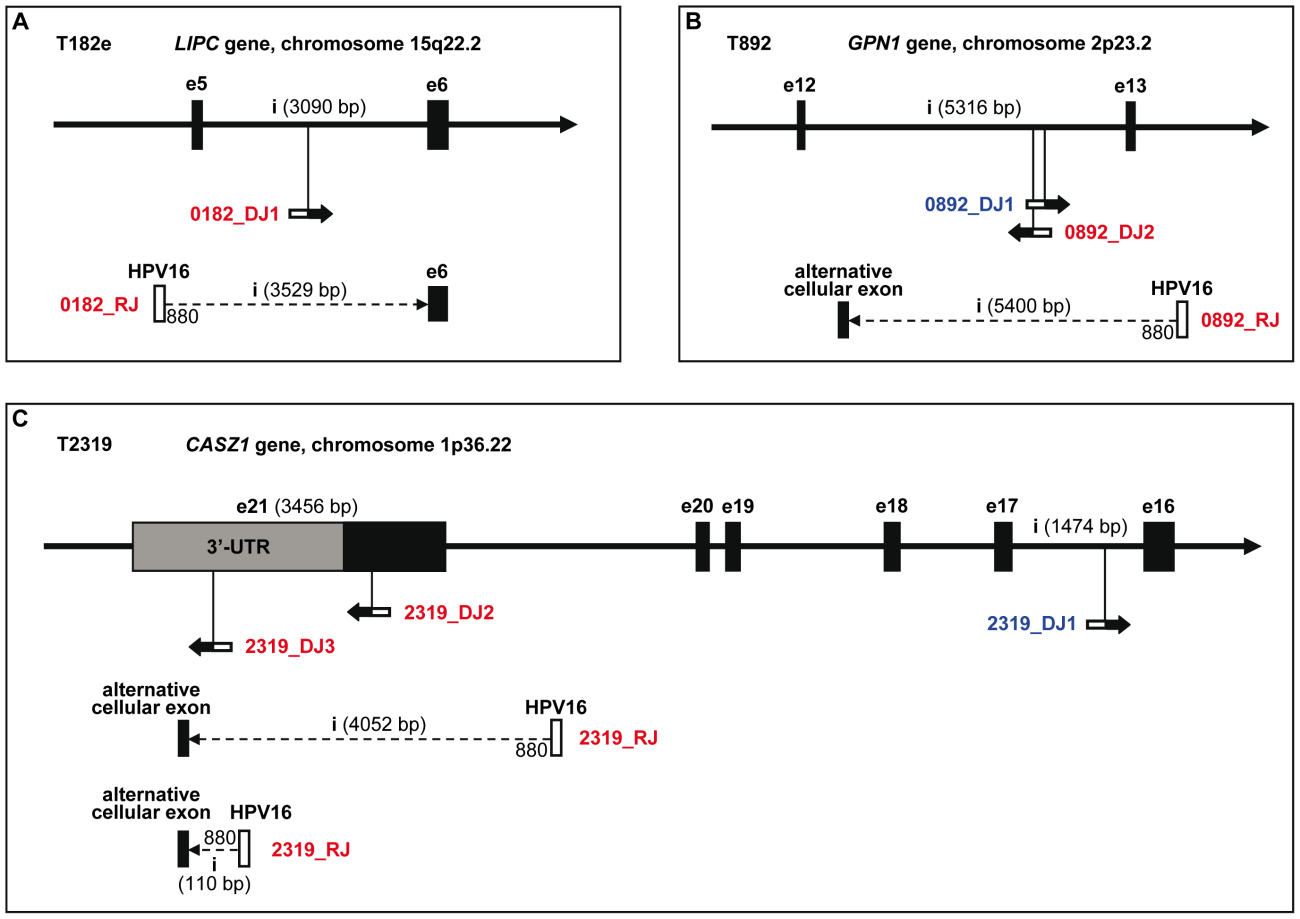
Examples of intragenic HPV16 DNA integration sites. (A) Tumor T182e has one HPV16 integration site 0182_DJ1, which is transcriptionally active (TA-group 1). The integrated HPV16 DNA is located in the intron (i) between exons (e) 5 and 6 of the cellular gene *LIPC* (transcript 003, Ensembl ID: ENST00000414170, plus strand, 10 exons), and has the same orientation as *LIPC*. APOT analysis identified an HPV16-cellular fusion transcript (0182_RJ) in which the viral exon is spliced to the downstream *LIPC* exon 6. (B) Tumor T892 (TA-group 2) has two HPV16 integration sites (0892_DJ1 and 0892_DJ2), which are both located in the intron between exons 12 and 13 of the cellular gene *GPN1* (transcript 001, Ensembl ID: ENST00000264718, plus strand, 14 exons). While 0892_DJ1 has the same orientation as *GPN1*, the transcriptionally active 0892_DJ2 has the opposite orientation. In the transcript 0892_RJ the viral exon is spliced to an alternative cellular exon. (C) In tumor T2319 (TA-group 2), all three identified HPV16 integration sites are located within the cellular gene *CASZ1* (transcript 003, Ensembl ID: ENST00000377022, 21 exons). Since the *CASZ1* gene is located on the minus strand, the sense orientation of the gene is from right to left. Junction 2319_DJ1 is located in an intron in opposite direction to *CASZ1*. Junctions 2319_DJ2 and 2319_DJ3 are located in the terminal exon 21 in the same direction as the *CASZ1* gene, DJ2 in the terminal part of the translated region and DJ3 in the 3′ untranslated region (3′-UTR). Both are possible templates for the HPV16-cellular fusion transcript 2319_RJ. – In the DJs and RJs, the open boxes denote the HPV16 part and the black boxes/arrows the fused cellular part. The arrow of the DJs indicates the sense orientation of the HPV16 oncogenes. Transcribed DJs and the RJs are shown in red, non-transcribed DJs in blue letters.

1) The viral-cellular DNA junctions (DJ) are sorted by chromosomal map position of the cellular sequences (fifth column).

2) u/r: u = unique cellular sequence; r = repetitive cellular sequence; the names of repeat sequences are given in parentheses.

3) t/d: t = gene directly targeted by HPV16 integration; d = the first gene located within 500 kb downstream of the DNA junction.

4) Orientation of the cellular gene with regard to the early region of integrated HPV16 DNA.

a) DNA junctions 2319_DJ1, DJ2 and DJ3 were discovered in the TEN16 sequence library by searching for DNA junctions located upstream of the identified RNA junction 2319_RJ (Table 2 and Figure 3). DJ2 contains a 65-bp sequence of chromosome 15 between HPV16 and chromosome 1 sequences. DJ1 and DJ3 were below the cutoff of 15 read pairs. DJ1 has also been identified by DIPS-PCR [44].

b) DNA junction 0892_DJ2 contains repetitive cellular sequence (AluSx), but could be assigned to chromosome 2 by taking into account the other DNA junction (0892_DJ1) and the RNA junction (0892_RJ; Table 2 and Figure 3).

c) DNA junctions 4426_DJ1 and 4024_DJ2 with <15 read pairs each were discovered by searching in the TEN16 sequence library for DNA junctions located upstream of the identified RNA junctions (Table 2).

d) DNA junction 3966_DJ1 was identified in the TEN16 sequence library after the second-round sequence-sorting.

e) DNA junction 2231_DJ1 was first mapped to two chromosome regions 4p16.3 and 4q35.2 with long identical sequences downstream of the junction. The mapping was then refined by long-range PCR to be on 4q35.2.

f) DNA junction CS_DJ1 with <15 read pairs was discovered by searching in the TEN16 sequence library for the known junction sequence [54].

g) DNA junction 4793_DJ3 with <15 read pairs was discovered by searching in the TEN16 sequence library for additional DNA junctions in the respective barcode.

h) Chromosome mapping of 4793_DJ1 and DJ2 was impossible because the flanking cellular sequences are composed mainly of the simple repeat sequence GGAAT.

### Comparison between HPV16-cellular DNA and RNA Junctions

APOT assay was performed for 46 of the 47 carcinomas analyzed by TEN16, the one exception being T1907U for which no RNA was available. The APOT RT-PCR technique can discriminate the episome-derived HPV oncogene transcript with a constant size of about 1 kb from integrate-derived HPV-cellular fusion transcripts with variable sizes (off-size transcripts) [21], as depicted in Figure 1B and 1C. From the 46 carcinomas, 22 HPV16-cellular fusion transcripts were determined in 22 samples giving one RNA junction per sample. Their junction features are summarized in Table 2 (for additional information see [50]).

**Table 2:** Comparison of HPV16-cellular DNA junctions and RNA junctions.

TA-		DNA junction (TEN16)		RNA junction (APOT)&			Distance	Distance
group	Tumor	ID	HPV16/cellular	ID	HPV16/cellular	Chr.	DJ/RJ§ (bp)	Do/Ac$ (bp)
**1** (1)	T18	0018_DJ1	3048/169306956	0018_RJ	0880/169309182	3	2226	4394
(n = 12)	T182e	0182_DJ1	2725/58836257	0182_RJ	0880/58837941	15	1684	3529a)
	T1875	1875_DJ1	3010/201823720	1875_RJ	0880/201823767	2	47	2177
	T2882	2882_DJ1	3596/146512049	2882_RJ	3596/146512049	2	0	n.a.
	T2967	2967_DJ1	2132/19610972	2967_RJ	0880/19611943	19	971	2223a)
	T3576	3576_DJ1	2350/44184762	3576_RJ	0880/44185187	6	425	1895
	T3966	3966_DJ1	2990/74540049	3966_RJ	0880/74542601	4	2552	4662
	T3987	3987_DJ1	3489/212251450	3987_RJ	3489/212251450	2	0	n.a.
	T4046	4046_DJ1	2146/73999476	4046_RJ	0880/73987773	13	11703	12969
	T4426	4426_DJ1#	2080/190056682	4426_RJ	0880/190044409	3	12273	13473
	T4601	4601_DJ1	0999/13858043	4601_RJ	0880/13860211	9	2168	2287
	T5234	5234_DJ1	2574/49319411	5234_RJ	0880/49318280	3	1131	2825a)
**2** (2)	T892	0892_DJ1	2970/27869352	no corresponding RJ		2	n.a.	n.a.
(n = 8)		0892_DJ2	2886/27869153	0892_RJ	0880/27865759	2	3394	5400
	T1509	1509_DJ1	2686/113482518	no corresponding RJ		8	n.a.	n.a.
		1509_DJ2	1228/33980798	1509_RJ	0880/33981987	9	1189	1537
	T2317	2317_DJ1	3667/230045578	no corresponding RJ		2	n.a.	n.a.
		2317_DJ2	1028/90729527	2317_RJ	1028/90729527	15	0	n.a.
	T2319	2319_DJ1#	4338/10707431	no corresponding RJ		1	n.a.	n.a.
		2319_DJ2#	3115/10699282	2319_RJ	0880/10697465	1	1817	4052
		2319_DJ3#	0910/10697545	2319_RJ	0880/10697465	1	80	110
	T3256	3256_DJ1	1642/182088476	no corresponding RJ		3	n.a.	n.a.
		3256_DJ2	2344/100058840	no corresponding RJ		10	n.a.	n.a.
		3256_DJ3	2612/100058865	3256_RJ	0880/100059016	10	151	1883
	T3427	3427_DJ1	1794/12233422	no corresponding RJ		12	n.a.	n.a.
		3427_DJ2	2461/19657318	3427_RJ	0880/19658664	17	1346	2927
	T4024	4024_DJ1	3018/17517026	no corresponding RJ		X	n.a.	n.a.
		4024_DJ2#	1841/100450200	4024_RJ	0880/100437501	4	12699	13660
	T5189	5189_DJ1	3286/25574739	no corresponding RJ		5	n.a.	n.a.
		5189_DJ2	2627/111272354	no corresponding RJ		8	n.a.	n.a.
		5189_DJ3	2353/128396523	5189_RJ	0880/128404749	8	8226	9699
**3** (3)	T186e*	0186_DJ1	2590/26947928	no corresponding RJ		9	n.a.	n.a.
(n = 2)		0186_DJ2	3897/30498221	no corresponding RJ		19	n.a.	n.a.
		no corresponding DJ		0186_RJ	0880/85394794	9	n.a.	n.a.
	T5066	5066_DJ1	2470/11159613	no corresponding RJ		20	n.a.	n.a.
		5066_DJ2	1813/61969363	no corresponding RJ		7	n.a.	n.a.
		no corresponding DJ		5066_RJ	0880/51086761	2	n.a.	n.a.

TA = TEN16/APOT comparison; DJ = DNA junction; RJ = RNA junction; Chr. = chromosome; n.a. = not applicable; Do = splice donor; Ac = splice acceptor.

(1) TA-group 1: samples with one DJ and a corresponding RJ.

(2) TA-group 2: samples with one corresponding DJ/RJ pair and additional DJs without RJ counterpart.

(3) TA-group 3: samples without corresponding DJ/RJ.

& The 22 RNA junctions (APOT) are part of a previous study [50] in which information on chromosomal locations, cellular genes and splicing is given, but without the exact position numbers of the cellular breakpoints.

§ Genomic distance between the cellular breakpoints of DJ and RJ.

$ Distance from the HPV16 splice donor (position 880) to the cellular splice acceptor (see Figure 1).

# Discovered by searching in the TEN16 sequence library for DJs located within 1 Mb upstream of the respective RJs.

* For sample T186e, the cellular sequences of DJ1 and RJ were both mapped to chromosome 9, but in opposite orientation to each other.

a) In the fusion transcript, the viral E6/E7 exon is spliced to the next downstream exon of the cellular gene (see Figure 3).

Comparison with the RNA junction sequences was performed to see which DNA junctions represent transcriptionally active HPV16 integrates. TEN16 DNA junctions corresponding to APOT RNA junctions were observed first in 16 samples. For the other six RNA junctions, the TEN16 sequence library was inspected again by searching for DNA junction sequences located within 1 Mb upstream of the RNA junction. Five DNA junctions from three samples could be identified (2319_DJs 1–3, 4024_DJ2 and 4426_DJ1), and all but 2319_DJ2 fell below the cutoff of 15 read pairs. For sample T3966, the DNA junction corresponding to the RNA junction was detected only after a second sequence-sorting under less stringent conditions (one mismatch per eight nucleotides allowed in the Nextera adapter). No corresponding DNA junctions, however, could be detected for the RNA junctions of tumors T186e and T5066.

Based on these results, the tumor samples with DNA/RNA junctions were classified into three TEN16/APOT (TA-) groups (see Table 2). TA-group 1 includes the samples (n = 12) specified by one DNA junction and a corresponding RNA junction (as an example for this group, the genomic integration site and fusion transcript structure of tumor T182e are shown in Figure 3A). Samples in TA-group 2 (n = 8) are featured by one DJ/RJ pair and additional DNA junctions (tumor T892 is shown as an example in Figure 3B). The sample T2319 is a unique case, in which two possible DNA counterparts (DJ2 and DJ3) for the same RNA junction were identified (Figure 3C). The two samples with unmatched DNA/RNA junctions constitute TA-group 3. Comparison between cellular DNA breakpoints and RNA exon boundaries revealed that in most cases (n = 17) the RNA junctions were created by splicing events, in which the viral donor (pos. 880) is fused to a cellular acceptor (depicted in Figure 1C, upper part). In three samples the DNA/RNA junctions are colinear (T2882, T3987 and T2317; see Table 2). T2882 and T3987 are cases in which the HPV16 breakpoints are located downstream of the splice acceptor at position 3358, and hence the viral-cellular junctions are maintained in the fusion transcripts (see Figure 1C, lower part). In 2317_DJ2 the HPV16 breakpoint is located at pos. 1028 indicating that the splice donor 880 was not used.

In 24 of the 46 carcinomas, the APOT analysis showed only the constant-size 1-kb HPV16 transcript assumed to be episome-derived (see Figure 1B). Nevertheless, 31 authentic DNA junctions of integrated HPV16 could be determined by TEN16 in 14 of the 24 samples. These samples were categorized as TA-group 4. This group includes six samples with one HPV16 integration site and eight samples with multiple integration sites ranging from two to six (Table 3). In ten other samples with constant-size HPV16 mRNA, no viral integration site could be identified by TEN16 (TA-group 5). Most likely, these cervical carcinomas contain truly episomal HPV16 DNA.

**Table 3:** Tumors with integrated HPV16 DNA, but without detected APOT fusion transcripts (TA-group 4).

Number of HPV 16 integrationsites (DJs) per tumor	Number of tumors (n = 14)	Tumor ID
1	6	T940, T1686, T2209,
		T2231, T2349, T2592
2	4	T2085, T2707, T3719,
		T4977
3	2	T4749, T4793
5	1	T841
6	1	T2548

**Figure 4:**
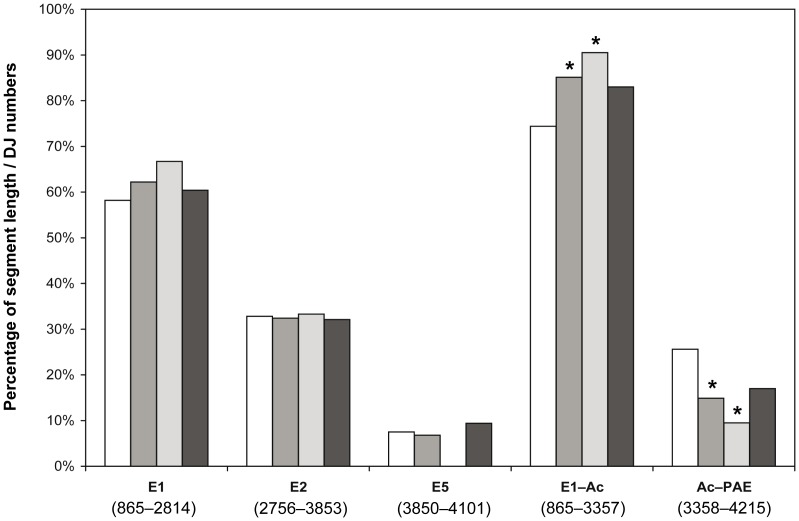
Frequency distribution of HPV16 3′-breakpoints in different segments of the HPV16 early region. The distribution of HPV16 3′-breakpoints of viral-cellular DNA junctions (n = 74) was analyzed within the five different segments E1, E2, E5, E1-Ac and Ac-PAE of the HPV16 early region. The positions of each segment in the HPV16 genome are given in parentheses. The E1-PAE segment of the HPV16 early region (pos. 865–4215, 3351 bp) was taken as reference. The relative length of each segment is shown by the white bars. The relative frequency of HPV16 3′-breakpoints within each segment is shown by the grey bars for all DNA junctions (DJ_all, n = 74, middle-grey bar), the transcribed DNA junctions (DJ_tr., n = 21, light-grey bar) and the non-transcribed DNA junctions (DJ_n.tr., n = 53, dark-grey bar). The exact two-tailed one-sample binomial test was used for statistical analysis by comparing the relative frequency of HPV16 3′-breakpoints in each segment to the relative segment length. Bars marked with asterisks indicate statistically significant results (P<0.05). Data are given in Table S4.

The HPV16 3′-breakpoints in the viral-cellular DNA junctions were analyzed for their distribution within different segments of the early region. The segment named E1-PAE extending from the E1 start at pos. 865 to the poly(A)-signal PAE at pos. 4215 (3351 bp) was taken as reference region. Seventy-four 3′-breakpoints are located within the E1-PAE region, and the only outlier in the L2 gene. The relative frequency of HPV16 3′-breakpoints within each segment was compared to the relative length of the segment. Statistical significance was calculated using the exact two-tailed one-sample binomial test. Comparison of the individual genes E1, E2 and E5 to the complete E1-PAE region revealed a frequency distribution of the 3′-breakpoints that is approximately proportional to the relative gene length (p>0.05 for difference). The E1-PAE region was then split into the two segments E1-Ac (2493 bp) and Ac-PAE (858 bp) located upstream and downstream, respectively, of the splice acceptor at position 3358. Here, a statistically significant (p<0.05) preferential distribution of the 3′-breakpoints in the E1-Ac segment became apparent, both when analyzing all 74 and the subset of 21 transcribed DNA junctions. The data are shown in Figure 4 and Table S4.

### Genomic Context of Integrated HPV16 DNA

For 73 of the 75 authentic DNA junctions, unequivocal mapping of the cellular sequence part to a specific chromosomal locus was possible (see Table 1). HPV16 DNA integration into a unique cellular sequence was observed in 60 cases. For the other 13 junctions, despite their repetitive nature the cellular parts could be assigned to specific chromosome regions based on sequence similarity. One exception was the repetitive cellular part of 0892_DJ2, for which the precise chromosome localization could only be unraveled by taking into account the sequence information of 0892_DJ1 and 0892_RJ (see Figure 3B). In this case, the localization was assured by additional junction-PCR and Sanger sequencing. The cellular sequence of 2231_DJ1 was first mapped to two potential locations 4p16.3 and 4q35.2, which are 99% identical to each other in a length of 6.7 kb immediately downstream of the junction. Long-range PCR with specific primers for each of the two regions identified 4q35.2 as the true integration site. For 4793_DJ1 and DJ2, mapping to a specific chromosome region was impossible because the cellular parts are composed mainly of GGAAT simple repeats.

For 36 DNA junctions in 22 samples, cellular genes were directly targeted by HPV16 DNA integration (see Table 1, column t/d). Three examples of intragenic HPV16 integration sites are shown in Figure 3. The targeted genes are in the same orientation as the integrated HPV16 E6/E7 in 16 cases, and in the opposite orientation in 20 cases (Table S5). The cellular DNA breakpoints are located in introns in 34 cases (see T182e and T892 in Figure 3A and 3B). The two exceptions with exon location are 2319_DJ2 and DJ3 (Figure 3C). The same orientation can lead to fusion mRNA in which the HPV16 E6/E7 exon is spliced to a genuine exon of the cellular gene. This was indeed the case for the three samples with known RNA junction sequences (T182e, T2967 and T5234), in which the HPV16 exon was spliced to the next downstream cellular exon (Figure 3A).

For tumor T2319 two novel DNA junctions were determined by TEN16, in addition to a DNA junction and the one RNA junction reported earlier [44]. The novel junctions DJ2 and DJ3 as well as the RNA junction are all located in sense orientation in the long 3′-terminal exon of the *CASZ1* gene, whereas the already known DNA junction DJ1 is located in antisense orientation in an intron (Figure 3C). Junction 2319_DJ2 has the peculiar structure that an intervening sequence of 65 bp mapping to chromosome 15 is located between HPV16 and the *CASZ1* sequence on chromosome 1. The tumor T892 is another noteworthy example. The two DNA junctions are both located in an intron of gene *GPN1*, but in opposite orientation to each other. The RNA junction (0892_RJ) corresponds to the DNA junction (0892_DJ2) with antisense orientation to *GPN1* (Figure 3B).

**Figure 5:**
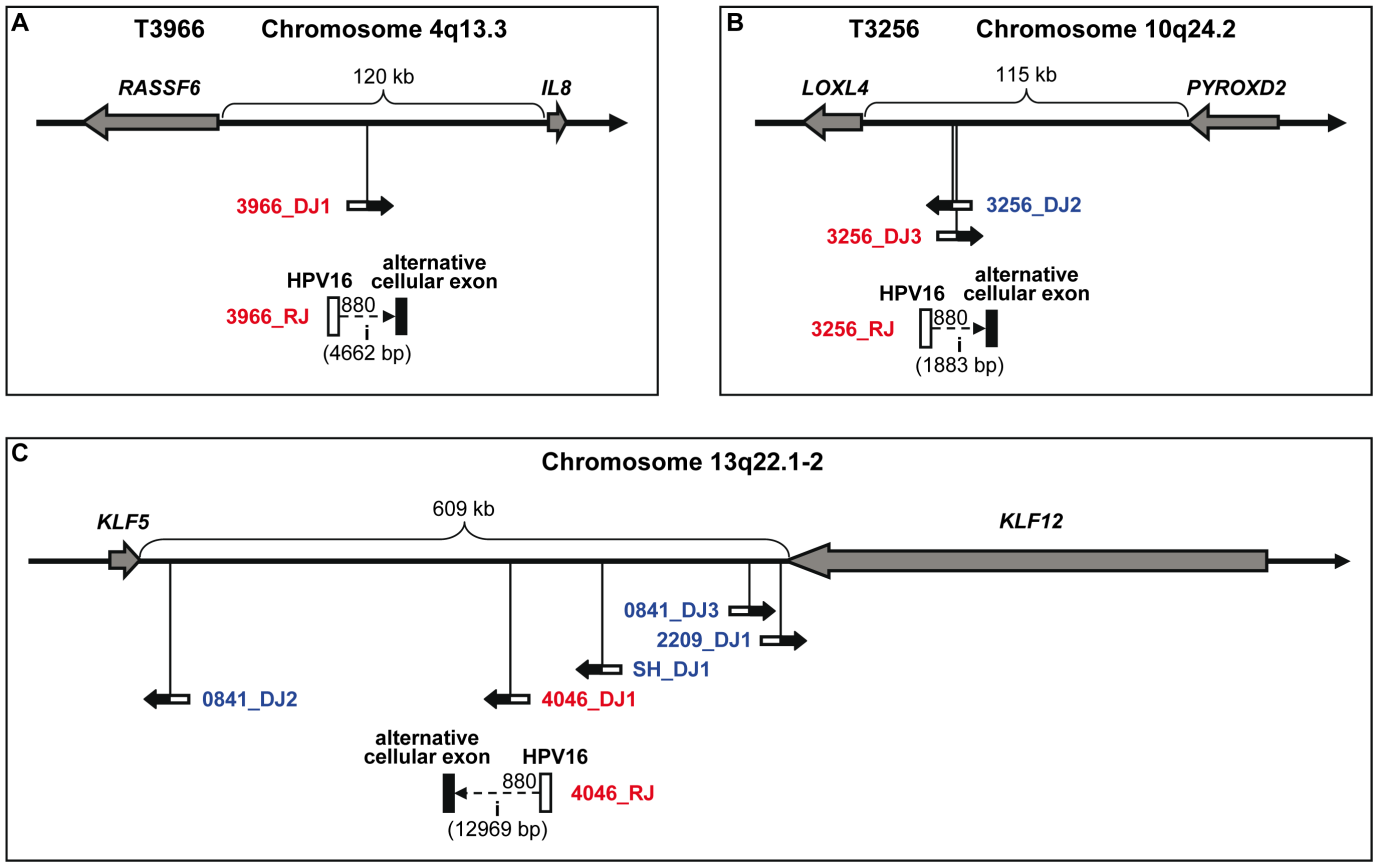
Examples of intergenic HPV16 DNA integration sites. (A) Tumor T3966 has one transcriptionally active HPV16 integration site 3966_DJ1 (TA-group 1), which is located between the cellular genes *RASSF6* and *IL8*, and has the same orientation as the downstream gene *IL8*. In the fusion transcript 3966_RJ, the viral exon is spliced to an intergenic alternative cellular exon. (B) Tumor T3256 (TA-group 2) has three HPV16 integration sites, one (3256_DJ1) on chromosome 3 (not shown) and two (DJ2 and DJ3) on chromosome 10q24.2 between the cellular genes *LOXL4* and *PYROXD2.* Only 3256_DJ3 is transcribed. The fusion transcript 3256_RJ is in opposite orientation to the downstream gene *PYROXD2*. (C) In the chromosome region 13q22.1-2, five HPV16 integration sites identified in four independent DNA samples are located in the large intergenic region between the cellular genes *KLF5* and *KLF12*. Only one integrated HPV16 DNA (4046_DJ1) is transcribed.

For the remaining 37 DNA junctions (of 26 samples) without directly targeted cellular gene, the genomic regions covering 500 kb downstream of the HPV16 integration sites were inspected. Examples of intergenic HPV16 integration sites are compiled in Figure 5. Cellular genes in the same orientation as HPV16 E6/E7 were found in 16 cases and in the opposite orientation in 13 cases (Table S6). In eight cases no downstream gene was detected in the 500-kb region (Table 1). In five of the 16 cases with identical orientation, RNA junction sequences have been determined. In all the five cases, splicing occurred to cellular sequences located before the downstream gene (T3966 is shown as an example in Figure 5A). Splicing to an exon of the downstream cellular gene was not observed.

**Figure 6:**
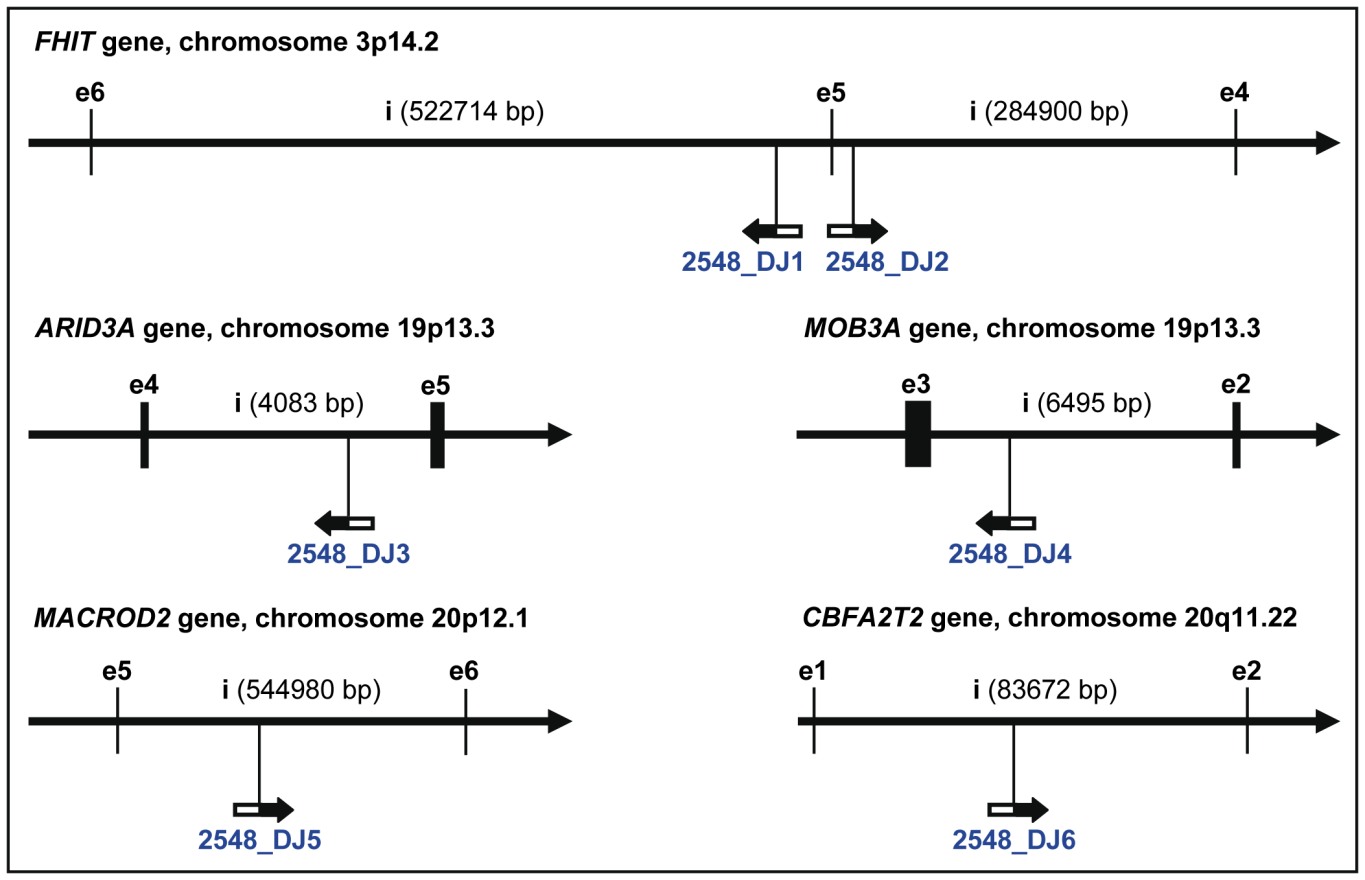
Tumor T2548 with six HPV16 DNA integration sites. The six integration sites are distributed on the chromosomes 3, 19 and 20 (two DJs on each). All six integration sites are intragenic, but none of them is transcriptionally active. The cellular genes directly targeted by HPV16 DNA integration in T2548 include *FHIT* (transcript 002, Ensembl ID: ENST00000468189, minus strand, 9 exons), *ARID3A* (transcript 001, Ensembl ID: ENST00000263620, plus strand, 9 exons), *MOB3A* (transcript 001, Ensembl ID: ENST00000357066, minus strand, 5 exons), *MACROD2* (transcript 010, Ensembl ID: ENST00000217246, plus strand, 17 exons) and *CBFA2T2* (transcript 003, Ensembl ID: ENST00000375279, plus strand, 12 exons).

Multiple HPV16 integration sites were determined in 20 samples (see Table S3). Concerning the samples with two integration sites (n = 12), the two DNA junctions are located in the same chromosome regions in three cases (T892 shown in Figure 3B, T2707 and MRI-H186) and on different chromosomes in nine cases. In the five samples with three integration sites, only one (T2319) has all the three cellular breakpoints close to each other (Figure 3C). From the four DNA junctions identified in CaSki, two are located on chromosome Xq27.3 at a distance of only 11.5 kb. In sample T841, the five integration sites are distributed over three chromosomes. And in sample T2548, the six integration sites are pairwise located on three different chromosomes (Figure 6). The most recurrent integration locus was a region of about 600 kb on chromosome 13q22.1-2 that harbors five HPV16 integration sites identified in four samples (SiHa, T841, T2209, T4046; see Figure 5C).

### HPV16 Variant Identification by E6 Sequencing

The HPV16 integration site analysis was combined with determination of the HPV16 variants present in the carcinoma samples. HPV16 variants can be classified based on nucleotide sequences of different genes [8], [55]–[57]. In this study the E6 gene was analyzed. An E6 segment of altogether 482 bp was amplified by two PCR reactions (pos. 111–383, pos.111–592) and tagged with sample-specific 5-nt barcodes. All E6-PCR products (47×2 = 94) were pooled and added to the TEN16 multiplex-PCR products for HiSeq2000 sequencing. After sorting by barcodes, the E6 sequences of all samples were compared to the published E6 sequences of different variants [8], [56], [57]. Among the 47 carcinomas, 23 harbor the European prototype (E-p; T350) and 22 samples the European T350G variant (E-T350G). Variants North-African 1 (NA1) and Asian-American (AA) were identified in one sample each. The results are summarized in Table 4. Within the European lineage, the E-T350G variant (E-L83V on the amino acid level) has been reported to be more prevalent than the E-p prototype (E-L83) in invasive cervical carcinoma and in cervical disease progression, depending on the analyzed population [58], [59]. In the present study, the distribution of tumors containing either integrated or episomal HPV16 DNA was compared between E-p and E-T350G. In the E-p group (n = 23), 20 cases contained integrated HPV16 DNA and 3 cases episomal HPV16 DNA (i.e. no viral-cellular DNA junctions have been identified by TEN16 analysis; see Table 4). The distribution in the E-T350G group (n = 22) was different because 6 cases contained episomal HPV16 DNA and 16 cases contained integrated HPV16 DNA. These differences, however, were not statistically significant (two-tailed P value = 0.28; Fisher’s exact test). The results indicate that the E-p and E-T350G variants of HPV16 are similar in their propensity to integrate.

**Table 4:** HPV16 E6 variants.

Lineage/sublineage	E6 nucleotide positions																Tumors*
	131	132	137	143	145	173	178	256	286	289	335	345	350	403	532	534	
HPV16REF	A	G	T	C	G	C	T	C	T	A	C	A	T	A	A	G	
E-p	–	–	–	–	–	–	–	–	–	–	–	–	–	–	–	–	T18, T186e, T892, T1686, T2317, T2548, T2967, T3042, T3315, T3427, T3576, T3966, T4024, T4046, T4426, T4601, T4793, T4977, T5189, T5446
	–	–	G	–	–	–	–	–	–	–	–	–	–	–	–	–	T2231
	–	–	–	–	–	T	–	–	–	–	–	–	–	–	–	–	T5066
	–	–	–	–	–	–	–	–	–	–	–	–	–	–	–	C	T2592
E-T350G	–	–	–	–	–	–	–	–	–	–	–	–	G	–	–	–	T182e, T707, T739, T940, T966, T1509, T1875, T1907U, T2085, T2209, T2319, T2620, T2882, T3256, T3719, T3799, T4749, T4755, T5234
	G	–	–	–	–	–	–	–	–	–	–	–	G	–	–	–	T2707
	–	–	–	–	–	–	–	T	–	–	–	–	G	–	–	–	T2349
	–	–	–	–	–	–	–	–	–	–	–	A/G	G	–	–	–	T841
NA1	–	–	–	–	T	–	–	–	A	G	T	–	G	–	–	–	T1520
AA	–	–	–	–	T	–	–	–	A	G	T	–	G	–	G	–	T3987
As	–	–	–	–	–	–	G	–	–	–	–	–	–	–	–	–	none
Af1	A/G	G/C	–	G	T	–	–	–	A	G	T	–	G	–	–	–	none
Af2	–	T	–	G	T	–	–	–	A	G	T	–	–	G	–	–	none

E-p = European prototype; E-T350G = European T>G at position 350; NA1 = North-American type 1; AA = Asian-American; As = Asian; Af1 = African type 1; Af2 = African type 2.

* Underlined are those tumors which seem to contain episomal HPV16 DNA, because no DNA junctions of integrated HPV16 DNA could be identified by the TEN16 analysis.

## Discussion

In this study we present the development of a novel multiplex strategy, TEN16, for analysis of HPV16 integration sites. The TEN16 procedure is a special form of targeted sequencing and exploits the high capacity of next-generation DNA sequencing. With this strategy, it was possible to identify 75 HPV16 integration sites (3′ DNA junctions) in a pooled analysis of DNA samples from 47 cervical carcinomas and 4 cell lines. High-quality nucleotide sequences of the DNA junctions were obtained.

Sample pooling was conducted twice during the whole TEN16 procedure. The first optional pooling reduced the number of Nextera transposition reactions from 50 to 10, and the second obligatory pooling combined all multiplex HPV16 PCR products into one mixture for Illumina HiSeq2000 paired-end sequencing. After data processing, a cutoff value of 15 read pairs per junction was introduced to pre-select the most promising junction candidates out of about 200,000 viral-cellular junction sequences for validation by junction-PCR. This measure proved to be very useful because it covered most validated DNA junctions (see Figure S1). For seven junctions with less than 15 read pairs each, identification was possible due to additional information.

The sensitivity of TEN16 can be estimated from the results obtained for the cervical carcinoma cell lines SiHa and CaSki. SiHa cells harbor a diploid set of one integrated HPV16 DNA copy, a situation similar to single-copy cellular genes [26], [27], [60]. SiHa DNA was mixed with four other carcinomas in the first pooling thus made up 1.8% of the final DNA mixture. The known 3′ junction (SH_DJ1) was present with 16 read pairs in the junction sequence library. CaSki cells contain an extremely high copy number (∼600) of integrated HPV16 full-length DNA arranged in head-to-tail concatemers and flanked by truncated terminal copies [26], [53]. This situation is similar to the presence of a small number of integrated viral genomes coexisting with a large number of episomes. Clusters of integrated HPV16 DNA with different copy numbers have been detected by *in situ* hybridization at 11 to 16 chromosomal sites in CaSki [27], [61], but there is only one transcriptionally active HPV16 integrate present at single-to-low copy number [27]. CaSki DNA was not subjected to the first pooling thus made up 9.1% (1/11^th^) of the final DNA mixture. Nonetheless, the known CaSki DNA junction, which is identical to the RNA junction [54], was present in the TEN16 library with only three read pairs (CS_DJ1) and would have escaped detection without the previous sequence information. Three novel CaSki DNA junctions were identified in our study. If CaSki DNA had been subjected to the first pooling, two of them (with read pair numbers of 28 and 42, respectively) might also have escaped detection. The three novel DNA junctions probably originate from non-transcribed concatemeric HPV16 integrates.

The overall outcome of the TEN16 study demonstrates that the concomitant analysis of HPV16 integration sites in a mixture of about 50 tumor samples is feasible. The high number of 75 validated HPV16 integration sites demonstrates the effective performance of this strategy. Nevertheless, for a sensitive detection of low-copy HPV16 integration sites, in particular against a high background of full-length HPV16 DNA, it seems appropriate for future TEN16 experiments to reduce the total number of DNA samples and, regarding the first pooling, either to waive it or to reduce the number of pooled samples.

Concerning the cervical carcinomas, 67 DNA junctions could be assigned to 37 of 47 samples (79%), while for ten samples no junction could be identified (21%). These percentages are in good agreement with the estimated ∼80% of HPV16-positive cervical carcinomas harboring integrated viral DNA [23]. Furthermore, 18 of the 37 carcinomas (49%) were found to contain more than one DNA junctions per sample. This is a high percentage not seen in previous studies, which reported multiple HPV16 integration sites in 15–20% of analyzed carcinomas [37], [45], [62]. These results might indicate the superior performance of TEN16 compared to the RS-PCR and DIPS-PCR methods for HPV16 integration site analysis.

Comparison of the DNA junctions with the 22 RNA junctions determined by APOT analysis allowed to identify the transcriptionally active HPV16 integration sites in 20 carcinomas (see Table 2). Two RNA junctions remained without corresponding DNA junctions for unknown reason. Twelve of the 20 carcinomas (60%) were found to contain a single transcriptionally active HPV16 integrate. The other 8 tumors (40%) are featured by a transcriptionally active HPV16 integrate together with one or two probably silent HPV16 integrates.

Strikingly, 31 DNA junctions could be identified in a subset of 14 tumor samples, for which the APOT analysis did not reveal any viral-cellular fusion transcript, but the purely viral oncogene mRNA (depicted in Figure 1B; see Table 3). These carcinomas would have been classified as containing episomal HPV16 DNA, if only the APOT data alone were taken into consideration. The validated DNA junctions contradict this interpretation. Our findings differ from an earlier study, in which the comparison of DIPS and APOT data led to the conclusion that most HPV-cellular junctions are actively transcribed [45]. Further transcriptional analysis in the 14 tumor samples based on the TEN16 data will clarify whether the 31 HPV16 integrates are indeed transcriptionally silent or transcribed into fusion transcripts not detected by APOT.

The HPV16 3′-breakpoints in the viral-cellular DNA junctions showed a statistically significant (p<0.05) preferential distribution within the early region segment that is located upstream of the splice acceptor at position 3358 and covers the complete E1 gene and the 5′-terminal half of the E2 gene (Figure 1, Figure 4 and Table S4). This uneven distribution supports the notion that one important aspect of integration-induced disruption of the HPV early region is to abolish production of the spliced purely viral early mRNAs and to replace them by viral-cellular fusion transcripts for deregulated E6/E7 oncogene expression [31]. It should be noted that 3′-breakpoint locations between the splice acceptor and the PAE signal also lead to viral-cellular fusion transcripts (see Figure 1C). Since the E2 gene is either decoupled or disrupted in all these cases, the results might also reflect the importance of E2 inactivation to release the integrated viral early promoter from E2-mediated repression [23]. Another possibility is that opening of the circular HPV genome for integration might occur with some predisposition within that region for the 3′-breakpoints. These different interpretations are not mutually exclusive.

HPV16 integration sites discovered in this study are located on almost all chromosomes, similar to the observations from other reports [28], [30], [37], [38], [50], [63]. Clustering of HPV16 integration sites was not particularly evident in our sample collection. The most recurrent integration locus was 13q22.1-2 with five integration sites of four samples (see Figure 5C). This chromosomal region contains the common fragile site FRA13C and has been identified as hotspot for HPV integration also in previous studies [28], [37]. Another known hotspot for HPV integration is region 8q24.21 in which the proto-oncogene *MYC* is located [41], [42]. The cell line MRI-H186 contains two DNA junctions and the carcinoma T5189 one DNA junction in this region.

Cellular genes are targeted by HPV16 integration in 36 of 73 cases (49%; see Table S5). These results support the previous observation that HPV integration has a preference for transcribed regions [28]. The integrated viral oncogenes and the targeted cellular genes have identical orientation in 16 cases. Fourteen of the 16 DNA junctions are located in introns. Preferential integration into introns, also observed by others [37], [45], [64], is most likely due to the much larger sizes of introns compared to exons. HPV integration into an intron will probably disturb expression of the targeted cellular gene. In a recent study, expression analysis was performed for ten tumors with cellular genes directly targeted by HPV DNA integration in the same orientation [44], including four samples (T182e, T2319, T2967 and T5234) also analyzed by TEN16 in the present study. In two of the ten cases (T2319 and T182e), the normal transcript of the affected cellular genes, *CASZ1* and *LIPC*, respectively, could not be detected. At least for sample T2319, the complete loss of *CASZ1* expression could be attributed to HPV16-induced insertional mutagenesis along with absence of the other allele not disrupted by integration [44]. Noteworthy, T2319 is the only example in our collection of carcinoma samples in which two HPV16 DNA junctions are located in an exon, here in the long 3′-terminal exon of the *CASZ1* gene (see Figure 3C). Altogether these examples support the assumption that insertional mutagenesis of cellular genes by HPV integration contributes to cervical carcinogenesis.

For another subset of HPV16 DNA junctions (n = 29), cellular genes could be found within 500 kb downstream of the HPV16 integration sites either in the same (n = 16) or in opposite (n = 13) orientation (see Table S6). In such cases it is not known whether and to which extent the integrated HPV16 DNA might affect expression of the flanking cellular genes. Since enhancers can activate genes located as far as 2–3 Mb off [65], influence of the HPV16 enhancer elements on neighboring cellular genes is possible.

Many cellular genes identified at or downstream of the 75 HPV16 integration sites are cancer genes, such as *MYC*, *ERBB2*, *FHIT*, *MECOM* (*EVI1*) and *BCAR4*. The search for cancer genes, using different approaches including sequencing of whole cancer genomes, has identified until now more than 400 human genes that are mutated in different types of cancer [66] (www.sanger.ac.uk/genetics/CGP/Census/). Applying the TEN16 integration site analysis to large series of additional cervical cancer and precancer samples will lead to a comprehensive mapping of HPV16 integration sites, far beyond the currently available data. This will allow to identify and to catalog precisely the hotspots of HPV16 integration as well as the affected cellular genes and the pathways in which they are involved. A database of viral integration sites related to human disease has recently been implemented [67], which will be helpful for a thorough interpretation of the data. HPV integration is a particular type of cancer mutation able to alter substantially the structure, expression and function of cellular genes. The identification of HPV integration sites in cervical carcinomas and other types of HPV-induced cancer therefore adds a special aspect to the cancer genome projects.

The TEN16 procedure, here established for identification of the potentially transcribed 3′ junctions of integrated HPV16 DNA, can be easily expanded to the determination of the 5′ junctions by selecting appropriate primers in the viral L1–L2 region. Since the cellular target sites of HPV integration are often affected by deletions and rearrangements [37], the determination of both the 3′ and 5′ junctions will convey a more complete picture of the integration effects on the viral and cellular genomes. Additional aspects of further development include the integration site analysis of other high-risk HPV types that will only need the selection of type-specific primer sets. The high capacity of NGS can be used further to address additional questions, like sequence variations in the HPV genome. As a prototypic example, the HPV16 E6 variants of the carcinoma samples have been determined concomitantly in the present study.

Although recurrent HPV insertion loci at the chromosomal level exist, the precise nucleotide sequences at the viral-cellular junctions are always different. Due to this feature, the viral-cellular chimeric sequences of integrated HPV genomes are unique fingerprints for every tumor. Therefore, the HPV integration sites have the potential to be used as personalized tumor biomarkers in the diagnosis, treatment monitoring and follow-up assessment of cervical carcinomas and high-grade precursor lesions in the individual patients. Molecular biomarkers for early detection of residual and recurrent disease will probably prove beneficial to improve treatment strategies and to increase survival [68]. A recent report demonstrates that viral-cellular junction sequences are specific markers which can be amplified from the circulating tumor DNA in cervical cancer patients [69]. The TEN16 strategy presented in this paper offers a new tool for efficient determination of HPV16 integration sites with precise nucleotide sequences that will help to exploit the potential of these unique molecular markers for prognostic evaluation and treatment of cervical cancer patients.

## Materials and Methods

### Ethics Statement

All patients provided written informed consent to use their biopsy material for further molecular analyses to be conducted in the Jena University Hospital and in collaboration with academic partners. This study was approved by the ethics committee of the Friedrich-Schiller University Jena (reference numbers 0175-02/00 and 2174-12/07).

### Clinical Samples and Cell Lines

All cervical carcinoma biopsies were taken from patients at the Department of Gynecology of Jena University Hospital, Germany, between 1995 and 2008. HPV genotype was determined by performing a multiplex real-time PCR detecting the seven most common high-risk HPV types [70]. Forty-seven HPV16-positive cervical carcinomas were identified for further analysis. The average age of these patients was 47 years within a range of 31 to 77 years. The clinical parameters were: 100% squamous cell carcinoma, 91% stage T1/T2, 60% N0 and 64% grade G1/G2. HPV16-positive human cervical carcinoma cell lines SiHa and CaSki were obtained from the American Tissue Culture Collection (ATCC), MRI-H186 and MRI-H196 from the DKFZ Tumorbank.

### DNA Reference Sequences

For the human genome, the latest major release GRCh37 was used as reference assembly (see http://www.ncbi.nlm.nih.gov/projects/genome/assembly/grc/human/). For HPV16, the HPV16REF sequence (7906 bp) was used which is based on the originally published sequence data [71] (GenBank accession number K02718; 7904 bp) plus revisions published by the Los Alamos National Laboratory from 1995 to 1997 in a compendium called “Human papillomaviruses: A compilation and analysis of nucleic acid and amino acid sequences”. The information is available in the Papillomavirus Episteme database [72]. HPV16REF differs from the NCBI reference sequence for HPV16 (accession number NC_001526.2; 7905 bp) in several positions. In the early region, HPV16REF has a G at position 1139 that is absent in NC_001526.2 and K02718).

### Nucleic Acid Isolation

High-molecular-weight genomic DNA was isolated from cell lines and clinical samples using the phenol-chloroform extraction method. The isolated DNA was roughly quantified by comparison of staining intensities with the λ/HindIII fragments (Invitrogen) in ethidium bromide-stained agarose gel. For RNA isolation, the tumor tissues were homogenized by using 0.55 mm-diameter injection needles. Total RNA was isolated using the NucleoSpin RNA II kit (Macherey-Nagel) with DNase treatment according to the protocol for tissue samples. RNA was quantified with the NanoDrop 1000 spectrophotometer.

### Amplification of Papillomavirus Oncogene Transcripts (APOT)

The APOT assay was performed basically as described [44], [50]. The gel-extracted RT-PCR products were sequenced at Seqlab (Göttingen, Germany).

### Nextera DNA Fragmentation and Tagging

First, five DNA samples of 10 ng each were pooled. The simultaneous DNA fragmentation and adapter tagging was performed with the Illumina-compatible Nextera DNA Sample Prep kit (Epicentre) according to the manufacturer’s instruction. Briefly, the pooled DNA of total 50 ng was mixed with 4 µl of 5× HMW buffer, 1 µl Nextera enzyme mix and water to a volume of 20 µl. After incubation at 55°C for 5 min, the Nextera fragments were purified with DNA clean & concentrator-5 spin column (Zymo) and eluted in 10 µl water. During column purification, transposase complexes were detached from DNA targets to expose the 9-nt single-stranded gaps accessible for subsequent modification.

### Blocking of the DNA 3′-ends

To minimize the whole-genome amplification of Nextera-treated DNA during PCR, the free 3′-OH ends upstream of the 9-nt gaps were blocked by enzymatic incorporation of ddNTP (see Figure 2B). Reaction components including 10 µl of purified Nextera fragments, 2.5 µl of 10× Klenow buffer, 1 µl ddNTP (1.25 mM each) and 0.5 µl Klenow exo- (5 U/µl; Fermentas) were mixed with water to 25 µl total, and incubated at 37°C for 15 min. The blocking reaction was heated at 90°C for 3 min to dissociate the 19-nt pMENTS oligonucleotide from the transposon ends, followed by incubation at 75°C for 5 min to renature the 5′-tagged dsDNA and then chilled at 4°C. The single-stranded pMENTS and impurities were eliminated by spin-column purification with DNA clean & concentrator-5. The purified DNA was eluted in 10 µl water.

### HPV16 DNA Enrichment by Multiplex PCR

The enrichment of HPV16-containing DNA was performed with the Multiplex PCR Plus kit (Qiagen) according to the user manual. For every Nextera-treated DNA pool, two multiplex PCR reactions were carried out in parallel. Briefly, 5 µl of purified 3′-blocked Nextera fragments were mixed with 25 µl of 2× Multiplex PCR master mix, 5 µl Q-solution, 2 µl of HPV16 primer mixture HPM-A or -B (5 µM of each primer; see Table S1), 2 µl of barcoded Nextera adapter (10 µM; see Table S2) and water to 50 µl total. Cycling conditions were: initial denaturation/activation at 95°C (5 min), 30 cycles including denaturation at 95°C (30 sec), annealing at 57°C (90 sec) and elongation at 72°C (40 sec). Equal volumes of all multiplex-PCR products were pooled and purified with illustra MicroSpin S-400 HR column (GE Healthcare).

### HPV16 E6 PCR

The E6 gene was used to classify HPV16 variants present in the clinical samples. To differentiate among the variants, nucleotide sequences at positions 131, 132, 143, 145, 178, 286, 289, 335, 350 and 532 of HPV16 are required [8], [56]. Two E6 PCR targets were designed to cover all the required positions, using a barcoded forward primer 5′-(5-nt barcode) AGGACCCACAGGAGCGAC-3′ (pos. 111–128) in combination with two reverse primers for each sample (5′-TGTTGTATTGCTGTTCTAATGTTG-3′, pos. 383–360; 5′-ATTCATGCAATGTAGGTGTATCTC-3′, pos. 592–569). PCR was performed with the FastStart High Fidelity kit (Roche). In a 50-µl reaction volume, 50 ng template DNA was mixed with 1×buffer, 2 mM MgCl_2_, 0.2 mM each dNTP, 0.4 µM forward primer, 0.4 µM reverse primer and 2.5 U enzyme mix. Cycling conditions were: initial denaturation at 95°C (2 min), 35 cycles including denaturation at 94°C (30 sec), annealing at 60°C (30 sec), and elongation at 72°C (30 sec). PCR products were purified with QIAquick PCR purification kit (Qiagen). Equal amount of all reactions were pooled and 10 ng was mixed with the TEN16 PCR products (1.4 µg) for HiSeq2000 sequencing.

### Next-generation Sequencing

High-throughput DNA sequencing was performed using the Illumina HiSeq2000 NGS technology. One microgram of the combined “TEN16+E6” PCR products was processed with the TruSeq DNA sample preparation kit (Illumina). The E-Gel SizeSelect system (Invitrogen) was used to collect adapter-ligated DNA fragments with insert sizes of 200–500 bp. The recovered DNA was loaded onto one lane of the HiSeq flow cell, at a concentration of 6.5 pM. A PhiX control v3 library (Illumina) was loaded onto another lane of the same run. Clonal amplification was done in cBot (Illumina) using the TruSeq paired-end v3 cluster generation chemistry (Illumina). For HiSeq2000 sequencing, the 200-cycle TruSeq-v3-SBS chemistry was used and 2×105 cycles of sequencing were carried out. Base-calling was conducted with Illumina’s RTA software version 1.10.36.

### Data Processing

Sequence read pairs were sorted into 58 barcodes based on the barcoded Nextera adapter primers for TEN16 (barcodes 01–11) and the barcoded E6 forward primers (barcodes 12–58) for variant analysis, using a custom script. In the first-round analysis, no mismatch was allowed. In the second-round analysis, one mismatch per eight nucleotides was allowed in the non-barcode area of the primers.

To identify potential HPV16-cellular junctions, the TEN16 read pairs were aligned to the combined “HPV16REF” and “GRCh37” human reference sequence first using BWA version 0.5.9-r16 [73] with default parameters. Mapped pairs with at least one read mapped to HPV16REF were extracted. Since BWA is unable to efficiently identify HPV16-cellular chimeric reads (see http://bio-bwa.sourceforge.net), many of them were reported as unmapped. Therefore, reads were re-mapped using BWA-SW version 0.5.9-r16 [74] with default parameters to identify chimeric reads containing both HPV16 and human sequences. Using custom scripts, four categories of mapped read pairs were identified from BWA and BWA-SW outputs: (1) pure HPV16 pairs, (2) pure cellular pairs, (3) pairs flanking HPV16-cellular junctions, and (4) pairs with at least one read containing the HPV16-cellular junction sequence (see Figure 2D). From categories 3 and 4, potential HPV16-cellular junctions were identified by visual inspection using a cutoff value of 15 read pairs.

To determine HPV16 variants, E6 read pairs were aligned to HPV16REF using BWA with default parameters. Only uniquely and properly mapped read pairs were selected for further analysis. “Properly mapped” were pairs with one read mapped to the sense strand and its mate mapped to the antisense strand of E6. Nucleotide polymorphisms in the E6 gene of individual tumor samples were called using SAMtools version 0.1.16-r963∶234 [75], and corrected manually by viewing the mapped reads in IGV [76]. Different HPV16 variants were assigned to the samples by unique nucleotide polymorphism patterns as reported [8], [56].

### Junction-PCR

PCR validation using an HPV16 primer and a cellular primer both specific for the same junction was conducted. The primer combinations are shown in Table S7. For every selected junction candidate, all the five DNA samples sharing the respective barcode were tested individually. The CaSki junctions were checked only with CaSki DNA. Junction-PCR was performed with the HotStarTaq Plus Master Mix kit (Qiagen). The 20-µl reaction components comprised 10 µl of 2x master mix, 1 µl HPV16 forward primer (5 µM), 1 µl cellular primer (5 µM), 10 ng template DNA, 2 µl of 10x CoralLoad and water. Cycling conditions were: initial denaturation/activation at 95°C (5 min), 35 cycles including denaturation at 94°C (30 sec), annealing at 59°C (30 sec), and elongation at 72°C (1 min). PCR products were purified with QIAquick PCR purification kit, and sequenced at GATC Biotech (Konstanz, Germany).

### Genomic Context Analysis

The junction sequences were subject to database searching for chromosome localization with the NCBI BLAST web interface (see http://blast.ncbi.nlm.nih.gov/Blast.cgi) using the MegaBLAST algorithm. The NCBI Map Viewer (see http://www.ncbi.nlm.nih.gov/mapview/) was used to map cellular breakpoints into specific chromosome regions, and to find out cellular genes located at or near the HPV16 integration sites. Cellular repetitive sequences were identified and classified by using the CENSOR tool [77]. The exon-intron structure of cellular genes was analyzed with the Ensembl genome browser (see http://www.ensembl.org/index.html).

### Statistical Analysis

Fisher’s exact test was used to analyze the association between HPV16 E6 variants E-p and E-T350G and the presence or absence of integrated HPV16 DNA in the tumors. The exact two-tailed one-sample binomial test was applied to analyze the frequency distribution of HPV16 3′-breakpoints in different segments of the HPV16 early region, always compared to the relative length of the segments. P values of 0.05 and lower were considered to be statistically significant.

### Accession Numbers

The dataset of Illumina HiSeq2000 read pairs has been deposited in the Sequence Read Archive (SRA) under the study accession number ERP002370. The nucleotide sequences of the 75 viral-cellular DNA junction sequences have been submitted to the European Nucleotide Archive (ENA). They have the accession numbers HE984501-HE984573, HE999548, and HF559481.

## Supporting Information

Figure S1 **Distribution of read pair numbers for the TEN16 junctions tested by junction-PCR.** Altogether 84 junctions (75 authentic as filled bars and 9 false-positive as open bars) are shown arranged by increasing numbers of read pairs, and for each junction a serial number was designated accordingly. The read pair numbers are shown in log scale. The dashed line indicates the cutoff level of 15 read pairs. Identification of the seven junctions below the cutoff is explained in the main text. For DNA junction 3966_DJ1 (serial number 1, labeled with asterisk), the first-round data analysis did not produce any read pair. In the second analysis at low stringency (see Materials and Methods), 155 read pairs of this junction were detected.
(TIF)

Table S1 **HPV16 forward primers for TEN16.**
(DOC)

Table S2 **Barcoded Nextera adapter sequence.**
(DOC)

Table S3 **HPV16-cellular DNA junctions validated by junction-PCR (sorted by sample ID).**
(DOC)

Table S4 **Frequency distribution of HPV16 3′-breakpoints of viral-cellular DNA junctions in different segments of the HPV16 early region.**
(XLS)

Table S5 **Cellular genes directly targeted by HPV16 DNA integration.**
(DOC)

Table S6 **First cellular genes within 500 kb downstream of integrated HPV16.**
(DOC)

Table S7 **Primer combinations for junction-specific PCR.**
(DOC)
