# Supplementary figures and images for: Multiplex Identification of Human Papillomavirus 16 DNA Integration Sites in Cervical Carcinomas
Source: PLoS One. 2013 Jun 18;8(6):e66693. doi: 10.1371/journal.pone.0066693 (PMC3688939; doi:10.1371/journal.pone.0066693)

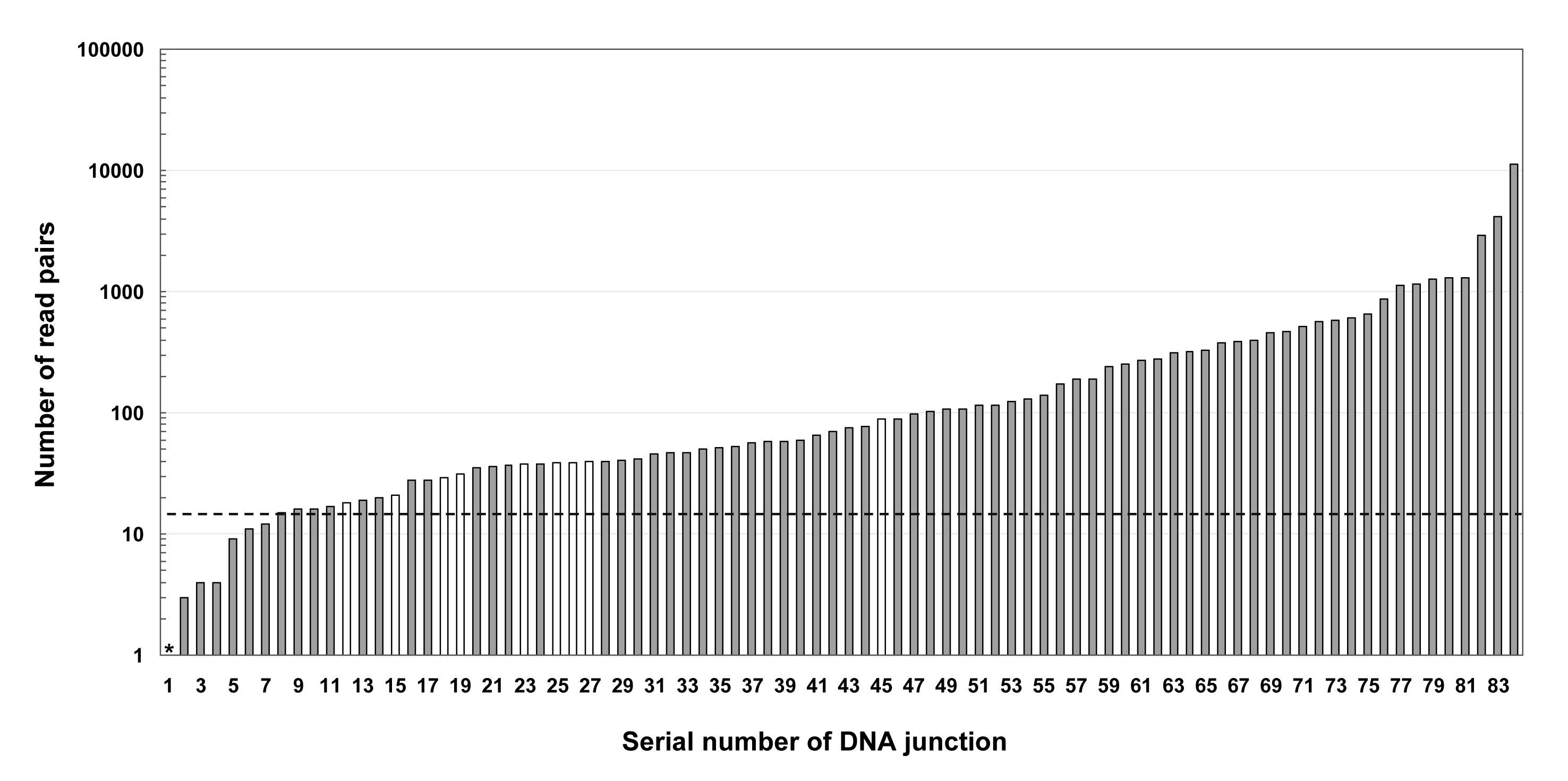

Supplement: Figure S1 — Distribution of read pair numbers for the TEN16 junctions tested by junction-PCR. Altogether 84 junctions (75 authentic as filled bars and 9 false-positive as open bars) are shown arranged by increasing numbers of read pairs, and for each junction a serial number was designated accordingly. The read pair numbers are shown in log scale. The dashed line indicates the cutoff level of 15 read pairs. Identification of the seven junctions below the cutoff is explained in the main text. For DNA junction 3966_DJ1 (serial number 1, labeled with asterisk), the first-round data analysis did not produce any read pair. In the second analysis at low stringency (see Materials and Methods), 155 read pairs of this junction were detected. (TIF) [file pone.0066693.s001.tif]
