# Supplementary material for: Multiplex Identification of Human Papillomavirus 16 DNA Integration Sites in Cervical Carcinomas
Source: PLoS One. 2013 Jun 18;8(6):e66693. doi: 10.1371/journal.pone.0066693 (PMC3688939; doi:10.1371/journal.pone.0066693)
Supplement: Table S1 — HPV16 forward primers for TEN16. (DOC) [file pone.0066693.s002.doc]

**Table S1. HPV16 forward primers for TEN16.**

| **HPV16 primer mix A (HPM-A)** | | **HPV16 primer mix B (HPM-B)** | |
| --- | --- | --- | --- |
| **Primer** | **Sequence (5’ – 3’)** | **Primer** | **Sequence (5’ – 3’)** |
| 16-878F | CAGGTACCAATGGGGAAGAG | 16-1080F | TGCACAGGAAGCAAAACAAC |
| 16-1276F | GAAGTGGAAACTCAGCAGATG | 16-1470F | TGCAAAGGCAGCAATGTTAG |
| 16-1672F | TCATGGGGAATGGTTGTG | 16-1865F | GAGACACGCCAGAATGGATAC |
| 16-2070F | AAAGGATTGTGCAACAATGTG | 16-2289F | ATATGGTGCAGCTAACACAGG |
| 16-2504F | TGGATGTAAAGCATAGACCATTG | 16-2702F | TCTCAAGGACGTGGTCCAG |
| 16-2883F | ATTACAAGGCCAGAGAAATGG | 16-3107F | GAAGTGCAGTTTGATGGAGAC |
| 16-3345F | CATCTGTGTTTAGCAGCAACG | 16-3542F | GACAGTGCTCCAATCCTCAC |
| 16-3762F | CATATGATAGTGAATGGCAACG | 16-4004F | CAGCAGCCTCTGCGTTTAG |
