# Supplementary material for: Multiplex Identification of Human Papillomavirus 16 DNA Integration Sites in Cervical Carcinomas
Source: PLoS One. 2013 Jun 18;8(6):e66693. doi: 10.1371/journal.pone.0066693 (PMC3688939; doi:10.1371/journal.pone.0066693)
Supplement: Table S2 — Barcoded Nextera adapter sequence. (DOC) [file pone.0066693.s003.doc]

**Table S2. Barcoded Nextera adapter sequence.**

| **Primer** | **Sequence (5’ – 3’)** |
| --- | --- |
| BNA01 | GTGAG-TCAGAGATGTGTATAAGAGACAG |
| BNA02 | GAGTG-TCAGAGATGTGTATAAGAGACAG |
| BNA03 | CTCAG-TCAGAGATGTGTATAAGAGACAG |
| BNA04 | CACTG-TCAGAGATGTGTATAAGAGACAG |
| BNA05 | CAGAG-TCAGAGATGTGTATAAGAGACAG |
| BNA06 | GACAG-TCAGAGATGTGTATAAGAGACAG |
| BNA07 | GAGAT-TCAGAGATGTGTATAAGAGACAG |
| BNA08 | GTCAT-TCAGAGATGTGTATAAGAGACAG |
| BNA09 | GAATG-TCAGAGATGTGTATAAGAGACAG |
| BNA10 | CATTG-TCAGAGATGTGTATAAGAGACAG |
| BNA11 | GGTAG-TCAGAGATGTGTATAAGAGACAG |
