# Supplementary material for: Multiplex Identification of Human Papillomavirus 16 DNA Integration Sites in Cervical Carcinomas
Source: PLoS One. 2013 Jun 18;8(6):e66693. doi: 10.1371/journal.pone.0066693 (PMC3688939; doi:10.1371/journal.pone.0066693)
Supplement: Table S3 — HPV16-cellular DNA junctions validated by junction-PCR (sorted by sample ID). (DOC) [file pone.0066693.s004.doc]

**Table S3. HPV16-cellular DNA junctions validated by junction-PCR (sorted by sample ID).**

|  |  | **HPV16** | **Cellular Sequence** | | | | **Cellular Gene** | | | **Accession** |
| --- | --- | --- | --- | --- | --- | --- | --- | --- | --- | --- |
| **DJ^1)^** | **Sample** | **breakpoint (ORF)** | **breakpoint** | **map** | **strand** | **u/r^2)^** | **name** | **t/d^3)^** | **orientation^4)^** | **Number** |
| 0018_DJ1 | T18 | 3048 (E2) | 169306956 | 3q26.2 | + | u | *MECOM* | t | opposite | HE984501 |
| 0182_DJ1 | T182e | 2725 (E1) | 58836257 | 15q22.2 | + | u | *LIPC* | t | same | HE984502 |
| 0186_DJ1 | T186e | 2590 (E1) | 26947928 | 9p21.2 | – | u | *IFT74* | t | opposite | HE984503 |
| 0186_DJ2 | T186e | 3897 (E5) | 30498221 | 19q11 | + | u | *URI1* | t | same | HE984504 |
| 0841_DJ1 | T841 | 2412 (E1) | 206103492 | 2q33.3 | – | r (AluSq) | *PARD3B* | t | opposite | HE984505 |
| 0841_DJ2 | T841 | 1953 (E1) | 73677253 | 13q22.1 | – | u | *KLF5* | d | opposite | HE984506 |
| 0841_DJ3 | T841 | 3281 (E2) | 74226231 | 13q22.2 | + | u | *KLF12* | d | opposite | HE984507 |
| 0841_DJ4 | T841 | 3413 (E2) | 24333382 | Xp22.11 | – | u | *FAM48B2* | d | same | HE984508 |
| 0841_DJ5 | T841 | 1703 (E1) | 24564835 | Xp22.11 | – | u | *PDK3* | d | opposite | HE984509 |
| 0892_DJ1 | T892 | 2970 (E2) | 27869352 | 2p23.2 | + | u | *GPN1* | t | same | HE984510 |
| 0892_DJ2 ^a)^ | T892 | 2886 (E2) | 27869153 | 2p23.2 | – | r (AluSx) | *GPN1* | t | opposite | HE984511 |
| 0940_DJ1 | T940 | 1529 (E1) | 124850317 | Xq25 | + | u | *DCAF12L2* | d | opposite | HE984512 |
| 1509_DJ1 | T1509 | 2686 (E1) | 113482518 | 8q23.3 | + | u | *CSMD3* | t | opposite | HE984513 |
| 1509_DJ2 | T1509 | 1228 (E1) | 33980798 | 9p13.3 | + | r (AluJb) | *UBAP2* | t | opposite | HE984514 |
| 1686_DJ1 | T1686 | 1354 (E1) | 57908866 | 17q23.2 | – | u | *VMP1* | t | opposite | HE984515 |
| 1875_DJ1 | T1875 | 3010 (E2) | 201823720 | 2q33.1 | + | u | *ORC2* | t | opposite | HE984516 |
| 1907_DJ1 | T1907U | 2412 (E1) | 62135042 | 14q23.2 | + | u | *HIF1A* | d | same | HE984517 |
| 2085_DJ1 | T2085 | 2774 (E1/E2) | 149157969 | 2q23.1 | + | u | *MBD5* | t | same | HE984518 |
| 2085_DJ2 | T2085 | 2198 (E1) | 11918118 | 16p13.13 | – | u | *BCAR4* | t | same | HE984519 |
| 2209_DJ1 | T2209 | 2637 (E1) | 74255979 | 13q22.2 | + | u | *KLF12* | d | opposite | HE984520 |
| 2231_DJ1 ^b)^ | T2231 | 1030 (E1) | 191040400 | 4q35.2 | – | r (MER31_I) | *DUX4L2* | d | opposite | HE984521 |
| 2317_DJ1 | T2317 | 3667 (E2) | 230045578 | 2q36.3 | + | u | *PID1* | t | opposite | HE984522 |
| 2317_DJ2 | T2317 | 1028 (E1) | 90729527 | 15q26.1 | – | u | *SEMA4B* | t | opposite | HE984523 |
| 2319_DJ1 ^c)^ | T2319 | 4338 (L2) | 10707431 | 1p36.22 | + | u | *CASZ1* | t | opposite | HE984524 |
| 2319_DJ2 ^c)^ | T2319 | 3115 (E2) | 10699282 | 1p36.22 | – | u | *CASZ1* | t | same | HE984525 |
| 2319_DJ3 ^c)^ | T2319 | 910 (E1) | 10697545 | 1p36.22 | – | u | *CASZ1* | t | same | HE984526 |
| 2349_DJ1 | T2349 | 2949 (E2) | 7521372 | Xp22.31 | – | u | *STS* | d | opposite | HE984527 |
| 2548_DJ1 | T2548 | 2868 (E2) | 60486966 | 3p14.2 | – | u | *FHIT* | t | same | HE984528 |
| 2548_DJ2 | T2548 | 1921 (E1) | 60535200 | 3p14.2 | + | u | *FHIT* | t | opposite | HE984529 |
| 2548_DJ3 | T2548 | 2019 (E1) | 963052 | 19p13.3 | – | u | *ARID3A* | t | opposite | HE984530 |
| 2548_DJ4 | T2548 | 3890 (E5) | 2080380 | 19p13.3 | – | u | *MOB3A* | t | same | HE984531 |
| 2548_DJ5 | T2548 | 2406 (E1) | 14886856 | 20p12.1 | + | r (AluSz) | *MACROD2* | t | same | HE984532 |
| 2548_DJ6 | T2548 | 3888 (E5) | 32116640 | 20q11.22 | + | u | *CBFA2T2* | t | same | HE984533 |
| 2592_DJ1 | T2592 | 1948 (E1) | 4583408 | 5p15.32 | – | u | *no gene* |  |  | HE984534 |
| 2707_DJ1 | T2707 | 3564 (E2) | 37818061 | 17q21.31 | + | u | *STARD3* | t | same | HE984535 |
| 2707_DJ2 | T2707 | 2970 (E2) | 37862307 | 17q21.31 | – | u | *ERBB2* | t | opposite | HE984536 |
| 2882_DJ1 | T2882 | 3596 (E2) | 146512049 | 2q22.3 | – | u | *no gene* |  |  | HE984537 |
| 2967_DJ1 | T2967 | 2132 (E1) | 19610972 | 19p13.11 | + | u | *GATAD2A* | t | same | HE984538 |
| 3256_DJ1 | T3256 | 1642 (E1) | 182088476 | 3q26.33 | + | u | *ATP11B* | d | same | HE984539 |
| 3256_DJ2 | T3256 | 2344 (E1) | 100058840 | 10q24.2 | – | u | *LOXL4* | d | same | HE984540 |
| 3256_DJ3 | T3256 | 2612 (E1) | 100058865 | 10q24.2 | + | u | *PYROXD2* | d | opposite | HE984541 |
| 3427_DJ1 | T3427 | 1794 (E1) | 12233422 | 12p13.2 | – | r (AluSg) | *BCL2L14* | t | opposite | HE984542 |
| 3427_DJ2 | T3427 | 2461 (E1) | 19657318 | 17p11.2 | + | u | *ULK2* | d | opposite | HE984543 |
| 3576_DJ1 | T3576 | 2350 (E1) | 44184762 | 6p21.1 | + | u | *SLC29A1* | d | same | HE984544 |
| 3719_DJ1 | T3719 | 2823 (E2) | 34896821 | 2p22.3 | – | r (L1M1_5) | *no gene* |  |  | HE984545 |
| 3719_DJ2 | T3719 | 2615 (E1) | 78848346 | 4q21.1 | – | u | *MRPL1* | t | opposite | HE984546 |
| 3966_DJ1 ^d)^ | T3966 | 2990 (E2) | 74540049 | 4q13.3 | + | u | *IL8* | d | same | HE984547 |
| 3987_DJ1 | T3987 | 3489 (E2) | 212251450 | 2q34 | + | u | *ERBB4* | t | opposite | HE984548 |
| 4024_DJ1 | T4024 | 3018 (E2) | 17517026 | Xp22.13 | + | u | *NHS* | t | same | HE984549 |
| 4024_DJ2 ^e)^ | T4024 | 1841 (E1) | 100450200 | 4q23 | – | u | *C4orf17* | t | opposite | HE984550 |
| 4046_DJ1 | T4046 | 2146 (E1) | 73999476 | 13q22.1 | – | u | *KLF5* | d | opposite | HE984551 |
| 4426_DJ1 ^e)^ | T4426 | 2080 (E1) | 190056682 | 3q28 | – | u | *CLDN1* | d | same | HE984552 |
| 4601_DJ1 | T4601 | 999 (E1) | 13858043 | 9p23 | + | u | *LINC00583* | d | same | HE984553 |
| 4749_DJ1 | T4749 | 2564 (E1) | 1493334 | 18p11.32 | + | r (L1) | *no gene* |  |  | HE984554 |
| 4749_DJ2 | T4749 | 1027 (E1) | 1506143 | 18p11.32 | – | u | *LINC00470* | d | same | HE984555 |
| 4749_DJ3 | T4749 | 3337 (E2) | 34242009 | 18q12.2 | + | u | *FHOD3* | t | same | HE984556 |
| 4793_DJ1 ^f)^ | T4793 | 1932 (E1) |  |  |  | r (GGAAT) |  |  |  | HE984557 |
| 4793_DJ2 ^f)^ | T4793 | 3881 (E5) |  |  |  | r (GGAAT) |  |  |  | HE984558 |
| 4793_DJ3 ^g)^ | T4793 | 1226 (E1) | 126291635 | 9q33.3 | + | u | *DENND1A* | t | opposite | HE999548 |
| 4977_DJ1 | T4977 | 2143 (E1) | 126782491 | 6q22.32 | – | r (LTR) | *CENPW* | d | opposite | HE984559 |
| 4977_DJ2 | T4977 | 3059 (E2) | 28835005 | 7p15.1 | – | u | *CREB5* | t | opposite | HE984560 |
| 5066_DJ1 | T5066 | 2470 (E1) | 11159613 | 20p12.2 | + | u | *no gene* |  |  | HE984561 |
| 5066_DJ2 | T5066 | 1813 (E1) | 61969363 | 7q11.21 | – | r (ALR1) | *no gene* |  |  | HF559481 |
| 5189_DJ1 | T5189 | 3286 (E2) | 25574739 | 5p14.1 | + | u | *no gene* |  |  | HE984562 |
| 5189_DJ2 | T5189 | 2627 (E1) | 111272354 | 8q23.2 | + | r (L1) | *no gene* |  |  | HE984563 |
| 5189_DJ3 | T5189 | 2353 (E1) | 128396523 | 8q24.21 | + | r (AluY) | *POU5F1B* | d | same | HE984564 |
| 5234_DJ1 | T5234 | 2574 (E1) | 49319411 | 3p21.31 | – | r (AluSc) | *USP4* | t | same | HE984565 |
| CS_DJ1 ^h)^ | CaSki | 3729 (E2) | 45659125 | 6p12.3 | – | u | *RUNX2* | d | opposite | HE984566 |
| CS_DJ2 | CaSki | 1973 (E1) | 11742450 | 10p14 | – | u | *USP6NL* | d | same | HE984567 |
| CS_DJ3 | CaSki | 975 (E1) | 144778296 | Xq27.3 | + | u | *SLITRK2* | d | same | HE984568 |
| CS_DJ4 | CaSki | 2987 (E2) | 144789749 | Xq27.3 | + | u | *SLITRK2* | d | same | HE984569 |
| MH186_DJ1 | MRI–H186 | 1224 (E1) | 128675817 | 8q24.21 | + | u | *MYC* | d | same | HE984570 |
| MH186_DJ2 | MRI–H186 | 2754 (E1) | 128746603 | 8q24.21 | + | u | *MYC* | d | same | HE984571 |
| MH196_DJ1 | MRI–H196 | 3858 (E5) | 47967861 | 11p11.2 | + | u | *PTPRJ* | d | same | HE984572 |
| SH_DJ1 | SiHa | 3133 (E2) | 74087562 | 13q22.1 | – | u | *KLF5* | d | opposite | HE984573 |

^1)^ DJ = DNA junction.

^2)^ u/r: u = unique cellular sequence; r = repetitive cellular sequence; the names of repeat sequences are given in parentheses.

^3)^ t/d: t = gene directly targeted by HPV16 integration; d = the first gene located within 500 kb downstream of the DNA junction.

^4)^ Orientation of the cellular gene with regard to the early region of integrated HPV16 DNA.

^a)^ DNA junction 0892_DJ2 contains repetitive cellular sequence (AluSx), but could be assigned to chromosome 2 by taking into account the other DNA junction (0892_DJ1) and the RNA junction (0892_RJ; Table 2 and Figure 3).

^b)^ DNA junction 2231_DJ1 was first mapped to two chromosome regions 4p16.3 and 4q35.2 with long identical sequences downstream of the junction. The mapping was then refined by long-range PCR to be on 4q35.2.

^c)^ DNA junctions 2319_DJ1, DJ2 and DJ3 were discovered in the TEN16 sequence library by searching for DNA junctions located upstream of the identified RNA junction 2319_RJ (Table 2 and Figure 3). DJ2 contains a 65-bp sequence of chromosome 15 between HPV16 and chromosome 1 sequences. DJ1 and DJ3 were below the cutoff of 15 read pairs. DJ1 has also been identified by DIPS-PCR [see reference 44].

^d)^ DNA junction 3966_DJ1 was identified in the TEN16 sequence library after the second-round sequence-sorting.

^e)^ DNA junctions 4024_DJ2 and 4426_DJ1 with <15 read pairs each were discovered by searching in the TEN16 sequence library for DNA junctions located upstream of the identified RNA junctions (Table 2).

^f)^ Chromosome mapping of 4793_DJ1 and DJ2 was impossible because the flanking cellular sequences are composed mainly of the simple repeat sequence GGAAT.

^g)^ DNA junction 4793_DJ3 with <15 read pairs was discovered by searching in the TEN16 sequence library for additional DNA junctions in the respective barcode.

^h)^ DNA junction CS_DJ1 with <15 read pairs was discovered by searching in the TEN16 sequence library for the known junction sequence [see reference 54].
