# Supplementary material for: Multiplex Identification of Human Papillomavirus 16 DNA Integration Sites in Cervical Carcinomas
Source: PLoS One. 2013 Jun 18;8(6):e66693. doi: 10.1371/journal.pone.0066693 (PMC3688939; doi:10.1371/journal.pone.0066693)
Supplement: Table S5 — Cellular genes directly targeted by HPV16 DNA integration. (DOC) [file pone.0066693.s006.doc]

**Table S5.** **Cellular genes directly targeted by HPV16 DNA integration.**

| **Targeted gene** | **Chr. map** | **DNA junction** | **Orientation^§^** |
| --- | --- | --- | --- |
| *CASZ1* | 1p36.22 | 2319_DJ2*^e)^ | same |
| *CASZ1* | 1p36.22 | 2319_DJ3*^e)^ | same |
| *GPN1* | 2p23.2 | 0892_DJ1 | same |
| *MBD5* | 2q23.1 | 2085_DJ1 | same |
| *FHIT* | 3p14.2 | 2548_DJ1 | same |
| *USP4* | 3p21.31 | 5234_DJ1*^i)^ | same |
| *LIPC* | 15q22.2 | 0182_DJ1*^i)^ | same |
| *BCAR4* | 16p13.13 | 2085_DJ2 | same |
| *STARD3* | 17q21.31 | 2707_DJ1 | same |
| *FHOD3* | 18q12.2 | 4749_DJ3 | same |
| *MOB3A* | 19p13.3 | 2548_DJ4 | same |
| *GATAD2A* | 19p13.11 | 2967_DJ1*^i)^ | same |
| *URI1* | 19q11 | 0186_DJ2 | same |
| *MACROD2* | 20p12.1 | 2548_DJ5 | same |
| *CBFA2T2* | 20q11.22 | 2548_DJ6 | same |
| *NHS* | Xp22.13 | 4024_DJ1 | same |
| *CASZ1* | 1p36.22 | 2319_DJ1 | opposite |
| *GPN1* | 2p23.2 | 0892_DJ2* | opposite |
| *ORC2* | 2q33.1 | 1875_DJ1* | opposite |
| *PARD3B* | 2q33.3 | 0841_DJ1 | opposite |
| *ERBB4* | 2q34 | 3987_DJ1* | opposite |
| *PID1* | 2q36.3 | 2317_DJ1 | opposite |
| *FHIT* | 3p14.2 | 2548_DJ2 | opposite |
| *MECOM* | 3q26.2 | 0018_DJ1* | opposite |
| *MRPL1* | 4q21.1 | 3719_DJ2 | opposite |
| *C4orf17* | 4q23 | 4024_DJ2* | opposite |
| *CREB5* | 7p15.1 | 4977_DJ2 | opposite |
| *CSMD3* | 8q23.3 | 1509_DJ1 | opposite |
| *UBAP2* | 9p13.3 | 1509_DJ2* | opposite |
| *IFT74* | 9p21.2 | 0186_DJ1 | opposite |
| *DENND1A* | 9q33.3 | 4793_DJ3 | opposite |
| *BCL2L14* | 12p13.2 | 3427_DJ1 | opposite |
| *SEMA4B* | 15q26.1 | 2317_DJ2* | opposite |
| *ERBB2* | 17q21.31 | 2707_DJ2 | opposite |
| *VMP1* | 17q23.2 | 1686_DJ1 | opposite |
| *ARID3A* | 19p13.3 | 2548_DJ3 | opposite |

DJ = DNA junction; Chr. = chromosome.

^§^ Orientation of the cellular gene with regard to the early region of integrated HPV16 DNA.

* DNA junctions with identified mRNA counterpart (Table 2).

*^e)^ HPV16 integration site is located in an exon.

*^i)^ HPV16 integration site is located in an intron. In the fusion transcript, the viral E6/E7 exon is spliced to the next downstream exon of the cellular gene.
