# Supplementary material for: Multiplex Identification of Human Papillomavirus 16 DNA Integration Sites in Cervical Carcinomas
Source: PLoS One. 2013 Jun 18;8(6):e66693. doi: 10.1371/journal.pone.0066693 (PMC3688939; doi:10.1371/journal.pone.0066693)
Supplement: Table S6 — First cellular genes within 500 kb downstream of integrated HPV16. (DOC) [file pone.0066693.s007.doc]

**Table S6. First cellular genes within 500 kb downstream of integrated HPV16.**

| **First gene downstream** | **Chr. map** | **DNA junction** | **Orientation^§^** |
| --- | --- | --- | --- |
| *ATP11B* | 3q26.33 | 3256_DJ1 | same |
| *CLDN1* | 3q28 | 4426_DJ1* | same |
| *IL8* | 4q13.3 | 3966_DJ1* | same |
| *SLC29A1* | 6p21.1 | 3576_DJ1* | same |
| *POU5F1B* | 8q24.21 | 5189_DJ3* | same |
| *MYC* | 8q24.21 | MH186_DJ1 | same |
| *MYC* | 8q24.21 | MH186_DJ2 | same |
| *LINC00583* | 9p23 | 4601_DJ1* | same |
| *USP6NL* | 10p14 | CS_DJ2 | same |
| *LOXL4* | 10q24.2 | 3256_DJ2 | same |
| *PTPRJ* | 11p11.2 | MH196_DJ1 | same |
| *HIF1A* | 14q23.2 | 1907_DJ1 | same |
| *LINC00470* | 18p11.32 | 4749_DJ2 | same |
| *FAM48B2* | Xp22.11 | 0841_DJ4 | same |
| *SLITRK2* | Xq27.3 | CS_DJ3 | same |
| *SLITRK2* | Xq27.3 | CS_DJ4 | same |
| *DUX4L2* | 4q35.2 | 2231_DJ1 | opposite |
| *RUNX2* | 6p12.3 | CS_DJ1 | opposite |
| *CENPW* | 6q22.32 | 4977_DJ1 | opposite |
| *PYROXD2* | 10q24.2 | 3256_DJ3* | opposite |
| *KLF5* | 13q22.1 | 4046_DJ1* | opposite |
| *KLF5* | 13q22.1 | SH_DJ1 | opposite |
| *KLF5* | 13q22.1 | 0841_DJ2 | opposite |
| *KLF12* | 13q22.2 | 0841_DJ3 | opposite |
| *KLF12* | 13q22.2 | 2209_DJ1 | opposite |
| *ULK2* | 17p11.2 | 3427_DJ2* | opposite |
| *PDK3* | Xp22.11 | 0841_DJ5 | opposite |
| *STS* | Xp22.31 | 2349_DJ1 | opposite |
| *DCAF12L2* | Xq25 | 0940_DJ1 | opposite |

DJ = DNA junction; Chr. = chromosome.

^§^ Orientation of the cellular gene with regard to the early region of integrated HPV16 DNA.

* DNA junctions with identified mRNA counterpart (Table 2).
