# Supplementary material for: Multiplex Identification of Human Papillomavirus 16 DNA Integration Sites in Cervical Carcinomas
Source: PLoS One. 2013 Jun 18;8(6):e66693. doi: 10.1371/journal.pone.0066693 (PMC3688939; doi:10.1371/journal.pone.0066693)
Supplement: Table S7 — Primer combinations for junction-specific PCR. (DOC) [file pone.0066693.s008.doc]

**Table S7. Primer combinations for junction-specific PCR.**

| **Junction** | **HPV16** | **Sequence (5’ – 3’)** | **Cellular** | **Sequence (5’ – 3’)** |
| --- | --- | --- | --- | --- |
| 0018_DJ1 | 16-2855F | AAACACATGCGCCTAGAATG | 169307093R | TGCAAAGCCTTGTGTCTCTG |
| 0182_DJ1 | 16-2571F | TGCTGGTACAGATTCTAGGTGG | 58836354R | CTCATCCCCAGGCTATTGTG |
| 0186_DJ1 | 16-2399F | CATTAGCAGATGCCAAAATAGG | 26947778F | AGCAAATACGGTGGTCCAAG |
| 0186_DJ2 | 16-3765F | ATGATAGTGAATGGCAACGTG | 30498413R | GGTTTGGAATGCGGTTTATG |
| 0841_DJ1 | 16-2166F | TGATGGAGGTGATTGGAAGC | 206103373F | TGGTGAAACCCCGTCTTTAC |
| 0841_DJ2 | 16-1861F | TATGGAGACACGCCAGAATG | 73677024F | CCCCTCCATGGTAAAGAATG |
| 0841_DJ3 | 16-3104F | GTGGAAGTGCAGTTTGATGG | 74226449R | TGTGGACCTGTCTGCCTTC |
| 0841_DJ4 | 16-3345F | CATCTGTGTTTAGCAGCAACG | 24333162F | GATATCCCAGGGTGCATCAC |
| 0841_DJ5 | 16-1564F | TGCGATTGGTGTATTGCTG | 24564744F | GGCCAGGACACTGTTACTTTG |
| 0892_DJ1 | 16-2933F | CCAACACTGGCTGTATCAAAG | 27869520R | ATTTGGTTGCAGGATTCCAC |
| 0892_DJ2 | 16-2739F | CGAGGACAAGGAAAACGATG | 27869118F | AGGCTGGTCTTGAACTCCTG |
| 0940_DJ1 | 16-1387F | AGTGGGGGAGAGGGTGTTAG | 124850570R | CTTCCTTCATTCCTGATTTTCC |
| 1509_DJ1 | 16-2648F | ACGAAAACGGAAATCCAGTG | 113482744R | TGGACAAGTCCCATTGTCAG |
| 1509_DJ2 | 16-1080F | TGCACAGGAAGCAAAACAAC | 33980911R | GTGCAGTGGTGTGATCTTGG |
| 1686_DJ1 | 16-1249F | AGCGAAGACAGCGGGTATG | 57908670F | CTACAGGCACTTGCCATCAC |
| 1875_DJ1 | 16-2855F | AAACACATGCGCCTAGAATG | 201823822R | AGATGGGAGGATTGCTTGAG |
| 1907_DJ1 | 16-2166F | TGATGGAGGTGATTGGAAGC | 62135165R | CTCATGCAGGGTAAGGTGTG |
| 2085_DJ1 | 16-2648F | ACGAAAACGGAAATCCAGTG | 149158135R | TTCATGTTGGATGGAGATTCC |
| 2085_DJ2 | 16-2053F | TCACAGGCAAAAATTGTAAAGG | 11917949F | ATCACTTGAACCCAGGAAGC |
| 2209_DJ1 | 16-2571F | TGCTGGTACAGATTCTAGGTGG | 74256010R | CCTACCCCATCCGATTTTG |
| 2231_DJ1 | 16-826F | ATTGTGTGCCCCATCTGTTC | 191040335F | TGAGTATACCATCTCCCAACATC |
| 2317_DJ1 | 16-3576F | CACACAAAGGACGGATTAACTG | 230045612R | GTCAGTCGGTCCTGAGAGATG |
| 2317_DJ2 | 16-826F | ATTGTGTGCCCCATCTGTTC | 90729424F | CCAATGTGCCTGGTTTATAGG |
| 2319_DJ1 | 16-4003F | ACAGCAGCCTCTGCGTTTAG | 10707552R | ACAACTCCCAGCCTGACAAG |
| 2319_DJ2 | 16-2855F | AAACACATGCGCCTAGAATG | 10699193F | CCTCGTCCTCGTCGTCTTC |
| 2319_DJ3 | 16-826F | ATTGTGTGCCCCATCTGTTC | 10697247F | ACAAAACTCCTTCCACAGACG |
| 2349_DJ1 | 16-2739F | CGAGGACAAGGAAAACGATG | 7521191F | ATCCCGCAGACAATAAGCAG |
| 2548_DJ1 | 16-2739F | CGAGGACAAGGAAAACGATG | 60486740F | AATTGCTTTTCCATCCCAAC |
| 2548_DJ2 | 16-1861F | TATGGAGACACGCCAGAATG | 60535506R | TTGTGTCTGACTTCCTGGTTG |
| 2548_DJ3 | 16-1861F | TATGGAGACACGCCAGAATG | 962851F | TAAACTGGTGTCCCGAGTGG |
| 2548_DJ4 | 16-3765F | ATGATAGTGAATGGCAACGTG | 2080231F | CACCACAGCATGAAGAGGAC |
| 2548_DJ5 | 16-2166F | TGATGGAGGTGATTGGAAGC | 14886936R | AACCCCTGACCTCAAGTGAC |
| 2548_DJ6 | 16-3765F | ATGATAGTGAATGGCAACGTG | 32116734R | AGCCCAAAACTTGGTACTGG |
| 2592_DJ1 | 16-1861F | TATGGAGACACGCCAGAATG | 4583384F | AGTTGCTTTTCTCCTCAAGACAG |
| 2707_DJ1 | 16-3443F | GGCACCGAAGAAACACAGAC | 37818232R | TTCCCAGCCCTCATACACTC |
| 2707_DJ2 | 16-2739F | CGAGGACAAGGAAAACGATG | 37862201F | GGTGATTCATCTCACCAGGAC |
| 2882_DJ1 | 16-3443F | GGCACCGAAGAAACACAGAC | 146511860F | GAAGCCCCTTCCCAATAAAG |
| 2967_DJ1 | 16-2053F | TCACAGGCAAAAATTGTAAAGG | 19611101R | CACCTGCAGCTTACATGCTC |
| 3256_DJ1 | 16-1564F | TGCGATTGGTGTATTGCTG | 182088683R | CCAGATTTAGTTCCACCCTTG |
| 3256_DJ2 | 16-2166F | TGATGGAGGTGATTGGAAGC | 100058725F | AAACCCACACCCGTATTTTG |
| 3256_DJ3 | 16-2571F | TGCTGGTACAGATTCTAGGTGG | 100059016R | AAGGTGTGTCTTCTCCTTGAGC |
| 3427_DJ1 | 16-1668F | ATGTTCATGGGGAATGGTTG | 12233225F | TTCCCAAATTCCTAGTCCTCTG |
| 3427_DJ2 | 16-2399F | CATTAGCAGATGCCAAAATAGG | 19657437R | AAGGAAAACACAAACCCTTCC |
| 3576_DJ1 | 16-2166F | TGATGGAGGTGATTGGAAGC | 44184846R | CCCTAACCCTTCTCCCTCAG |
| 3719_DJ1 | 16-2648F | ACGAAAACGGAAATCCAGTG | 34896656F | ATCAGGCTCCCAGATCTTACC |
| 3719_DJ2 | 16-2571F | TGCTGGTACAGATTCTAGGTGG | 78848012F | CTTGGCCTCCTAAAGTGCTG |
| 3966_DJ1 | 16-2855F | AAACACATGCGCCTAGAATG | 74540287R | AACATTAGGTCAGGAGGACAGC |
| 3987_DJ1 | 16-3345F | CATCTGTGTTTAGCAGCAACG | 212251596R | CGAGACAAACCCAAACAAGG |
| 4024_DJ1 | 16-2855F | AAACACATGCGCCTAGAATG | 17517275R | CCACTGCCACCAGAAACTG |
| 4024_DJ2 | 16-1668F | ATGTTCATGGGGAATGGTTG | 100449923F | TCACTTTCATGGGAATGGTATG |
| 4046_DJ1 | 16-2053F | TCACAGGCAAAAATTGTAAAGG | 73999318F | ACATCTGGGGGTCTGTTTTG |
| 4426_DJ1 | 16-1861F | TATGGAGACACGCCAGAATG | 190056604F | TGGATTTGAGAAGTGTGAGTGG |
| 4601_DJ1 | 16-826F | ATTGTGTGCCCCATCTGTTC | 13858226R | TGATTTGCTCCACAGTCCAG |
| 4749_DJ1 | 16-2399F | CATTAGCAGATGCCAAAATAGG | 1493494R | AGGGCCAGACTGATTCACAG |
| 4749_DJ2 | 16-826F | ATTGTGTGCCCCATCTGTTC | 1506006F | AACATGCCTTTTATTCCAGACC |
| 4749_DJ3 | 16-3104F | GTGGAAGTGCAGTTTGATGG | 34242094R | ACATATGCGCTTGAGGTGTG |
| 4793_DJ1 | 16-1668F | ATGTTCATGGGGAATGGTTG | 4793-1-C | TCCATTCCATTCCATTCCAC |
| 4793_DJ2 | 16-3765F | ATGATAGTGAATGGCAACGTG | 4793-2-C | GGAATGGAATTGAATGGAATG |
| 4793_DJ3 | 16-1080F | TGCACAGGAAGCAAAACAAC | 126291858R | TTCACGTTCTTTTGGCTGTC |
| 4977_DJ1 | 16-1861F | TATGGAGACACGCCAGAATG | 126782395F | TGGGAGTTTCCCTACACACG |
| 4977_DJ2 | 16-2855F | AAACACATGCGCCTAGAATG | 28834796F | TCCCCACACCCTTCTGTTAC |
| 5066_DJ1 | 16-2399F | CATTAGCAGATGCCAAAATAGG | 11159811R | GCTATTGTGCCCTGGCTTAG |
| 5066_DJ2 | 16-1668F | ATGTTCATGGGGAATGGTTG | 61969281F | CTGCTTGTTATGTCTGCAAGTG |
| 5189_DJ1 | 16-3104F | GTGGAAGTGCAGTTTGATGG | 25574913R | CAAGGGTTATTTGGATGTTGTG |
| 5189_DJ2 | 16-2399F | CATTAGCAGATGCCAAAATAGG | 111272474R | AGGTCCTTCACATCCATTGC |
| 5189_DJ3 | 16-2166F | TGATGGAGGTGATTGGAAGC | 128396646R | CCACCATGCCCTCTAATTTC |
| 5234_DJ1 | 16-2399F | CATTAGCAGATGCCAAAATAGG | 49319251F | CTGGCCAACATGGTAAAACC |
| CS_DJ1 | 16-3545F | AGTGCTCCAATCCTCACTGC | 45658930F | AAGGTTTTCATTGGCTGCTG |
| CS_DJ2 | 16-1861F | TATGGAGACACGCCAGAATG | 11742161F | TGTCTCCCCAAAATTCATACG |
| CS_DJ3 | 16-826F | ATTGTGTGCCCCATCTGTTC | 144778518R | TTCCCCAACCCCATTATACC |
| CS_DJ4 | 16-2739F | CGAGGACAAGGAAAACGATG | 144789921R | GAGCTCCTGTTCACCAAACC |
| MH186_DJ1 | 16-1080F | TGCACAGGAAGCAAAACAAC | 128676044R | ACCCATCAAATCCCTCTGC |
| MH186_DJ2 | 16-2648F | ACGAAAACGGAAATCCAGTG | 128746768R | TTGTGTTATTCCACGGCATG |
| MH196_DJ1 | 16-3765F | ATGATAGTGAATGGCAACGTG | 47967951R | ATGCTTTTGCCTCAGACAGC |
| SH_DJ1 | 16-2933F | CCAACACTGGCTGTATCAAAG | 74087417F | ATCTGAGGTGTCCTTCATTGG |
